# An Effective Oral Drug Delivery Route for Pharmacokinetic Complications: Spirulina Lipid Nanotechnology System

**DOI:** 10.1002/advs.202509731

**Published:** 2025-09-12

**Authors:** Jiahui Ye, Xinyi Wu, Jin Liu, Yongmi Guo, Chong Ji, Zhouyue Wang, Di Yang, Min Zhou

**Affiliations:** ^1^ Eye Center The Second Affiliated Hospital Zhejiang University School of Medicine Hangzhou 310000 P. R. China; ^2^ Institute of Translational Medicine Zhejiang University Hangzhou 310029 P. R. China; ^3^ Zhejiang University‐University of Edinburgh Institute (ZJU‐UoE Institute) Zhejiang University Haining 314400 P. R. China; ^4^ Research Center for Life Science and Human Health, Binjiang Institute of Zhejiang University Zhejiang University Hangzhou 310053 P. R. China; ^5^ Zhejiang University‐Ordos City Etuoke Banner Joint Research Center Zhejiang University Haining 314400

**Keywords:** drug delivery, dysregulated gastrointestinal motility, irritable bowel syndrome, liposome, spirulina

## Abstract

Irritable bowel syndrome (IBS) is a prevalent gastrointestinal disorder characterized by mental manifestations, abdominal pain, and alterations in defecation habits. The incidence rate in some areas even exceeds 20%. Secondary to chronic, recurrent gastrointestinal motility dysfunction, for patients with IBS, upon oral administration of drugs, the fluctuation of bioavailability is typically far more significant than that of healthy individuals. Nevertheless, at present, few studies have put forward targeted drug delivery systems addressing this aspect. In this study, an oral spirulina nanotechnology System (SP@TIIAn) is developed that integrates tanshinone IIA liposome with spirulina for enhanced IBS treatment. Owing to the passive targeting of intestinal villi and enhanced adhesion by spirulina and nanoparticles, it is discovered that, in contrast to enteric‐coated capsules, this system is more beneficial for guaranteeing the pharmacokinetic stability of IBS. SP@TIIAn effectively treats multiple gut‐brain symptoms of IBS, contributing to providing new alternatives for the development of clinical medications for IBS.

## Graphical abstract

Spirulina‐nano system facilitates the formation of drug coating on spirulina's surface through electrostatic adsorption. It targets intestinal villi effectively and overcomes the pharmacokinetic complications of IBS‐D. This system successfully carried out the treatment of multiple symptoms, including diarrhea, visceral hypersensitivity, mental symptoms, etc. This provides a reference for the development of drug delivery systems for different disease subtypes.

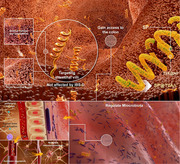

## 1 Introduction

Irritable bowel syndrome (IBS) is a highly prevalent gastrointestinal disorder affecting 7–21% of the global population, often resulting in diminished quality of life.^[^ ^1^, ^2^ ^]^ Beyond gastrointestinal issues, up to 50% of patients also experience neurological comorbidities such as anxiety and visceral hypersensitivity, which are frequently linked to dysfunction of the gut‐brain axis, thereby complicating the clinical presentation.^[^ ^3^, ^4^, ^5^ ^]^ While the precise etiology of IBS remains elusive, factors like gut microbiota dysbiosis, altered intestinal barrier integrity, and stress have been identified as potential contributors.^[^ ^6^, ^7^ ^]^ Current treatment strategies for IBS are predominantly palliative, addressing symptoms rather than underlying pathophysiological mechanisms. These treatments are often limited by systemic side effects or disruption of the microbiota, underscoring the urgent need for novel and more effective therapeutic approaches.^[^ ^8^ ^]^ The pathophysiological complexity of IBS, particularly in diarrhea‐predominant subtypes (IBS‐D), poses significant challenges for effective oral drug delivery. Patients with IBS‐D frequently experience accelerated intestinal transit, which diminishes the time available for oral medications to be absorbed in the small intestine, thereby reducing bioavailability and therapeutic efficacy.^[^ ^9^ ^]^ Moreover, the gastrointestinal inflammation, dysbiosis, and impaired barrier function of IBS‐D further impair their absorption, distribution, and bioavailability. This altered pharmacokinetic environment hinders conventional drug formulations from achieving therapeutic levels, resulting in suboptimal clinical outcomes.^[^ ^10^ ^]^ Unfortunately, no effective strategy has yet addressed the drug absorption challenges in IBS‐D patients. Although advanced drug delivery systems, such as controlled‐release formulations and nanoparticles, have been investigated, they still fail to reliably enhance bioavailability in the altered intestinal environment of IBS‐D, rendering effective oral drug delivery a significant challenge.^[^ ^11^, ^12^ ^]^

Tanshinone IIA (TIIA), a bioactive compound derived from Salvia miltiorrhiza, exhibits anti‐inflammatory, antioxidant, and vasodilatory properties, which can help alleviate symptoms such as visceral hypersensitivity and intestinal inflammation.^[^ ^13^, ^14^ ^]^ However, its therapeutic utility is limited by poor aqueous solubility, rapid hepatic metabolism, and insufficient intestinal retention.^[^ ^15^ ^]^ Although various drug delivery strategies, including encapsulation and liposomal formulations, have been employed to enhance the stability and bioavailability of TIIA, these methods still present deficiencies when confronted with IBS‐D.^[^ ^16^ ^]^

Given the pathophysiological state of IBS‐D, an ideal drug delivery system for IBS‐D should remain unaffected by the dysfunctional gut environment, thereby stabilizing the patient's pharmacokinetics. *Spirulina platensis* (*S. platensis*, SP) is a spiral‐shaped microalgae, and its drug delivery capabilities have been fully developed through genetic engineering, nanotechnology, and other means.^[^ ^17^, ^18^, ^19^ ^]^ Moreover, SP has garnered attention for its potential health benefits, particularly in supporting gut health. Rich in essential nutrients, antioxidants, and prebiotic fibers, spirulina has been shown to enhance gut microbiota diversity, improve intestinal barrier function, and modulate immune responses.^[^ ^20^, ^21^ ^]^ These properties make it a promising candidate for the treatment of IBS‐D and position spirulina as a viable drug delivery vehicle for oral administration. How to further transform SP to enable it to cope with the pharmacokinetic complications of IBS‐D is the key point of this research.

The use of spirulina as a drug carrier presents several challenges, and one of the main limitations is the inability to control the release of encapsulated drugs, which can result in premature drug release and reduced therapeutic efficacy. In response to these challenges, we have developed an oral microalgae‐nano drug delivery system (SP@TIIAn) that combines SP with TIIA liposome to effectively treat IBS‐D. This system enhances the adhesion to intestinal tissues through a viscous antibacterial nanocoating and further targets the intestinal villi by integrating the unique helical structure of SP. Furthermore, the encapsulation of liposomes enhances the solubility, bioavailability, and acid resistance of TIIA. SP@TIIAn provides a multi‐dimensional therapeutic modality, including intestinal and mental symptoms, and offers thoughts for the development of drug delivery systems targeted at specific disease subsets (Movie S1, Supporting Information).

## 2 Results and Discussion

### 2.1 Synthesis and Characterization

**Figure 1:**
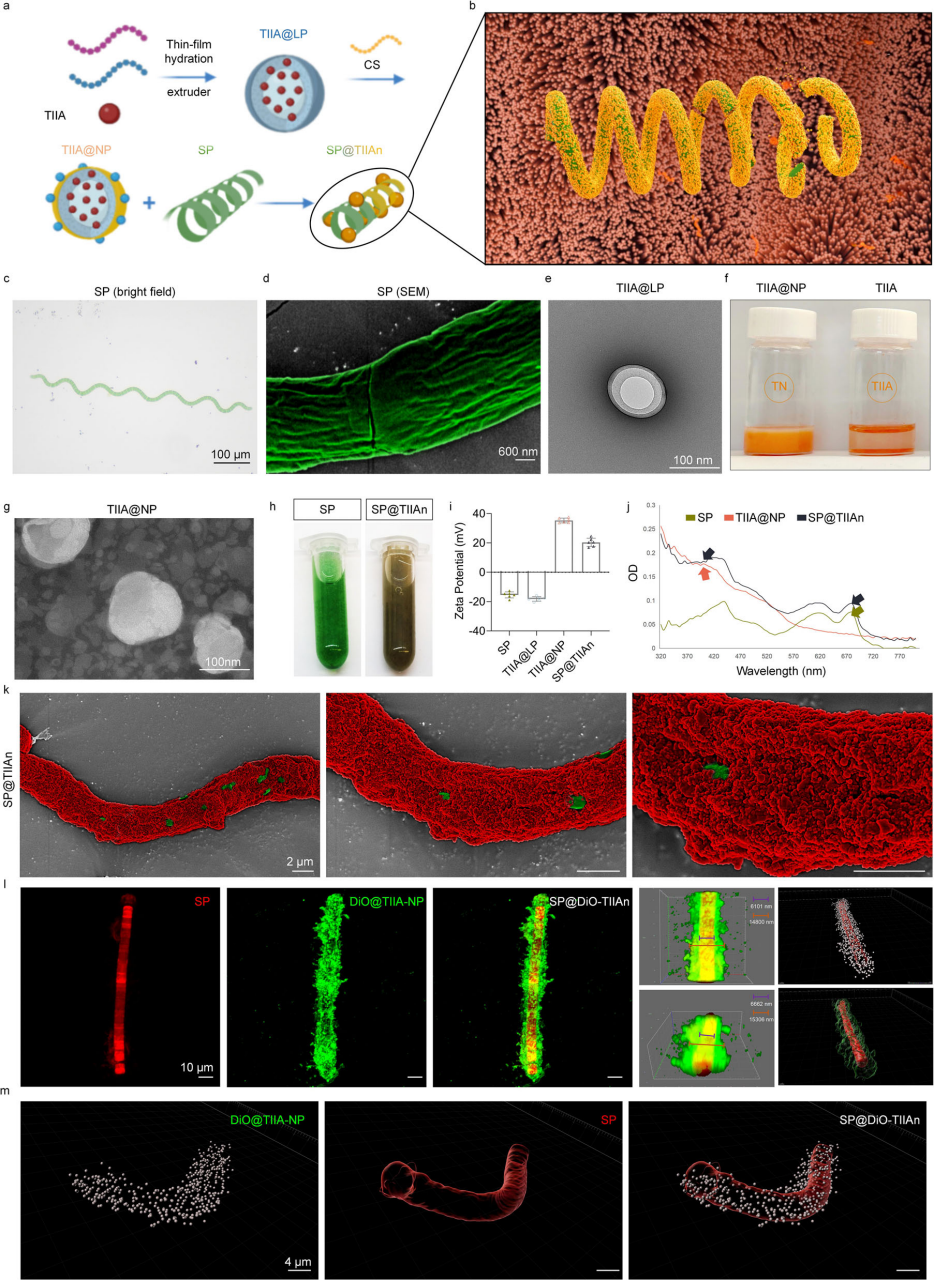
Synthesis and characterization of SP@TIIAn. a) Synthesis route of SP@TIIAn. Created in BioRender. Ji, C. (2025) https://BioRender.com/aws4y45. b) Schematic Diagram of the SP@TIIAn. c) Bright field microscopy, and SEM (pseudo‐color) images of SP. d,e) TEM image of TlIA@NP. f) Dissolution of TlIA@NP and TIIA. g) Hydrodynamic size distribution of TIIA@LP and TlIA@NP. h) Photographs of SP and SP@TIIAn. i) Zeta potential values (*n* = 6). j) UV–vis absorption spectra of TlIA@NP, SP, and SP@TIIAn. k) Pseudo‐color SEM image of SP@TIIAn. SP (green) and TlIA@NP (red). l,m) The fluorescence, depth‐encoded, and 3D reconstruction.

Given the limited water solubility of TIIA, we encapsulated it within liposomes by the thin‐film hydration method, thereby forming TIIA@LP. These nanodroplets were subsequently coated with a positively charged, viscous antibacterial chitosan (CS) layer, resulting in TlIA@NP. This engineered nanoparticle system was then integrated with SP by adhering TlIA@NP onto its cell membrane (**Figure** **1**a,b). SP displayed a green appearance and maintained a characteristic helical structure with a rough surface (Figure 1c,d; Figure S1, Supporting Information). A liposome extruder was employed to further homogenize TlIA@NP. TlIA@NP significantly improved the solubility and dispersibility of TIIA, overcoming its inherent low solubility. Transmission electron microscopy (TEM) analysis and scanning electron microscopy (SEM) revealed monodisperse nanoparticles with uniform spherical morphology. After being homogenized by extrusion devices, the diameters of both TIIA@LP and TIIA@NP can be ≈100 nm (Figure 1e–g). The electrostatic interaction between TlIA@NP (+37.5 mV) and SP (−15.5 mV) facilitates the rapid and uniform assembly of TlIA@NP onto the extensive surface area of SP, resulting in a positively charged biological system (SP@TIIAn) (Figure 1h). UV–vis spectroscopy confirmed the successful assembly by detecting characteristic absorption peaks of both TlIA@NP and SP. SEM further verified the comprehensive and uniform coating of SP with TlIA@NP, thereby supporting its potential as an advanced drug delivery platform (Figure 1i–k; Figure S2, Supporting Information). SP exhibits distinct fluorescence (red) due to its high chlorophyll content, making it a promising candidate as an imaging biomaterial.^[^ ^22^ ^]^ This fluorescence also confirms that the active components of SP remain intact following assembly, thus laying the foundation for its prebiotic activity. To assess drug loading, we labeled TlIA@NP with the membrane dye DiO (green) and performed 3D scanning. The diameter of SP was found to be ≈6101 nm, while the nanocoating thickness was ≈14800 nm, highlighting the biosystem's excellent drug‐loading capacity. This point can be identified in SPs under various growth conditions (Figure 1l,m; Figure S3, Supporting Information). Subsequent experiments determined that optimal encapsulation efficiency (EE) was achieved when the reaction time was 60 min (Figure S3, Supporting Information).

### 2.2 Biodistribution Profile, Adhesion Properties, and Anti‐SIBO Effects

**Figure 2:**
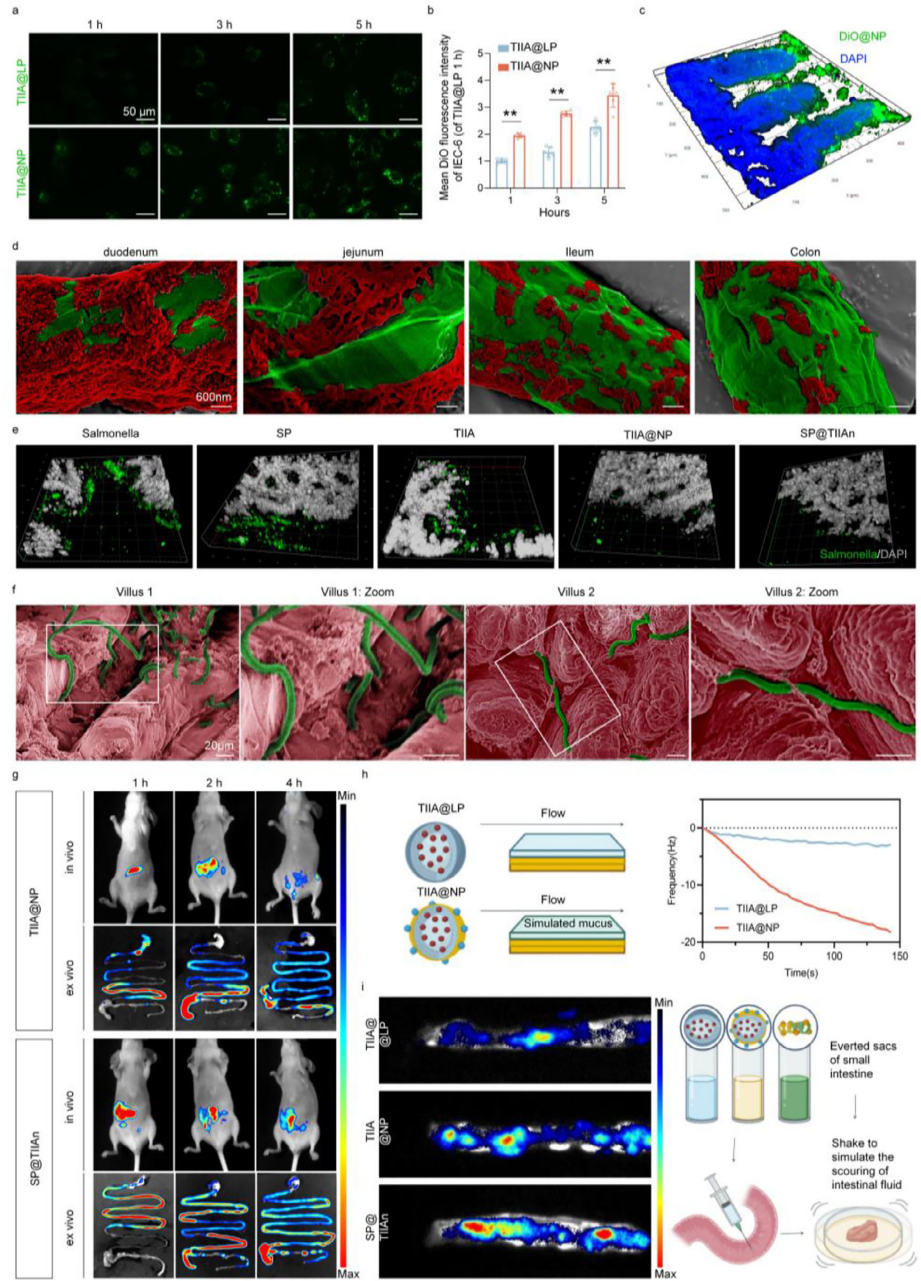
The biodistribution characteristics of SP@TIIAn. a) Fluorescence images and a) intensity of IEC‐6 incubated with DiO‐labeled TlIA@NP and TIIA@LP. c) Intestinal frozen section after oral administration of DiO‐TlIA@NP. d) SEM images (pseudo‐color) of SP@TIIAn in the small intestine. e) Immunofluorescence staining of *Salmonella* in the intestine. f) SEM (pseudo‐color) of the small intestine after oral administration with SP. g) In vivo fluorescence imaging of mice at different time points after oral administration with TlIA@NP and SP@TIIAn (DiO channel). h) QCM frequency change curves of TlIA@NP and TIIA@LP upon introduction into the MUC2 coating. Created in BioRender. Ji, C. (2025) https://BioRender.com/aws4y45. i) Fluorescence images of DiO‐labeled nanoparticles, which attach to the everted sacs after oscillation and shaking. Created in BioRender. Ji, C. (2025) https://BioRender.com/aws4y45. Data are presented as means ± SD (*n* = 6), ^**^ represents *p* < 0.01.

To confirm the adhesion of TlIA@NP to intestinal tissue, DiO‐labeled TlIA@NP and TIIA@LP were co‐incubated with intestinal epithelial cells (IEC‐6). The TlIA@NP group exhibited significantly higher cell membrane fluorescence compared to TIIA@LP, a trend similarly observed in intestinal tissue. This close adhesion of TlIA@NP to the intestinal mucus layer can be attributed to the electrostatic attraction between the positively charged nanoparticles and the negatively charged mucus (**Figure** **2**a–c; Figure S4, Supporting Information).^[^ ^23^ ^]^ SEM analysis of the intestinal contents confirmed that the in vivo release kinetics of SP@TIIAn were similar to those observed in vitro, revealing a gradual release of TlIA@NP in the intestine (Figure 2d; Figure S5, Supporting Information). For the potential concurrent SIBO in IBS‐D, we used Salmonella enterica Typhimurium SL1344 as a representative pathogen.^[^ ^24^ ^]^ SP@TIIAn maintained the effectiveness of TIIA in treating small intestinal bacterial overgrowth (SIBO) and exhibited significant antibacterial activity, primarily attributed to the CS coating (Figure 2e; Figure S6, Supporting Information). Subsequent SEM analysis of SP in different locations of the small intestine revealed its elongated helical structure, which intricately interlocks within the crevices of intestinal villi. This unique morphology not only facilitates prolonged retention of SP in the intestine but also enhances SP's potential to resist excessive motility and secretion in IBS‐D (Figure 2f). To test this potential, TlIA@NP labeled with DiO was used to synthesize SP@TIIAn, allowing us to investigate the distribution and retention of the drug. Fluorescence excitation in both the red channel for SP and the green channel for DiO enabled clear visualization (Figure S7, Supporting Information). After gavage, the overlapping fluorescence distribution of both signals indicated strong binding between TlIA@NP and SP. In vivo fluorescence images demonstrated that SP@TIIAn exhibited significantly greater signal strength and longer duration, suggesting prolonged intestinal retention of TIIA (Figure 2g; Figure S8, Supporting information). Quartz crystal microbalance (QCM) analysis was conducted to further evaluate the surface properties.^[^ ^25^ ^]^ Suspensions of TIIA@LP and TlIA@NP were introduced into the chamber, where the key intestinal mucus protein Mucin 2 was spin‐coated onto the chip surface to simulate the intestinal mucus layer. Changes in frequency (ΔF) indicated the adhesion mass, revealing significantly higher adhesion of the modified TlIA@NP compared to TIIA@LP (Figure 2h). This result is consistent with the conclusion of co‐incubation with intestinal epithelial cells. Finally, we used everted small intestine sacs coated with TIIA@LP, TlIA@NP, or SP@TIIAn. The sacs were oscillated and incubated to simulate intestinal peristalsis and scouring associated with IBS‐D diarrhea. The results demonstrated that the SP assembly markedly enhanced the distribution and intensity of the DiO signal, indicating a specific interaction between SP and the intestinal villi (Figure 2i).

### 2.3 Ability to Address Pharmacokinetic Disorders

**Figure 3:**
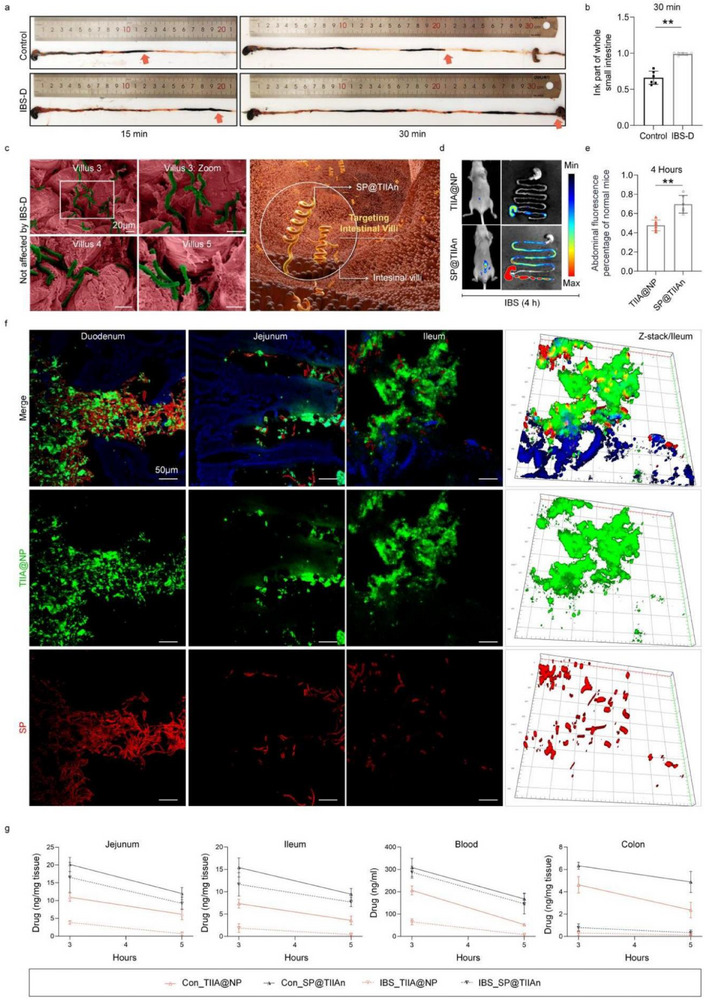
SP@TIIAn mitigates pharmacokinetic disorders associated with IBS‐D. a) Ink propulsion experiment and b) the distance traveled by the ink in 30 min (*n* = 6). c) SEM images (pseudo‐color) of the small intestine tissue in IBS‐D mice treated with SP. d) Fluorescence images and e) abdominal fluorescence intensity in IBS‐D mice treated with TlIA@NP (DiO) and SP@TIIAn (DiO) after 4 h gavage (*n* = 6). f) Frozen sections of duodenum, jejunum, and ileum in IBS‐D mice after oral administration of SP@TIIAn. g) Drug contents in the jejunum, lleum, blood, and colon after different treatments (*n* = 3). Data are presented as means ± SD, ^**^ represents *p* < 0.01.

Individuals with IBS‐D typically experience altered small intestinal motility, characterized by increased peristalsis and disorganized contractions, leading to inadequate drug dissolution and affecting drug absorption and metabolism in the small intestine.^[^ ^26^ ^]^ Intestinal peristalsis was evaluated by intragastric administration of Indian ink, and the results demonstrated that the ink moved significantly faster in the IBS‐D model compared to the control group, confirming the presence of the studied symptoms (**Figure** **3**a,b). This uncoordinated intestinal motility can lead to diarrhea, including IBS‐D, which may disrupt the biodistribution of drugs. To investigate whether SP could retain its efficacy, we conducted SEM analysis of intestinal tissue in the context of IBS‐D. The results revealed that both the digestive metabolism of SP and its interaction with the villi in the small intestine remained unaffected (Figure 3c; Figure S9, Supporting Information). Despite the presence of diarrhea, in vivo imaging demonstrated that SP@TIIAn remains largely unaffected by IBS‐D. The fluorescence intensity in the abdominal region was significantly higher in the SP@TIIAn group compared to TlIA@NP, indicating more extensive and stable drug distribution (Figure 3d,e; Figure S10, Supporting Information). Further examination of intestinal segments via frozen sectioning revealed that the co‐localization of SP and TlIA@NP was most prominent in the duodenum and gradually decreased along the intestine (Figure 3f). Moreover, TIIA levels in blood and different segments of the intestine of mice were quantified using liquid chromatography‐tandem mass spectrometry (LC‐MS/MS), and the results exhibited that TlIA@NP failed to prevent the significant reduction in drug content caused by IBS‐D. In contrast, oral administration of SP@TIIAn demonstrated similar pharmacokinetic behavior in both the IBS‐D and control groups, not disturbed by symptoms of diarrhea. This property was not exhibited in the colon and should be ascribed to the absence of intestinal villi therein (Figure 3g; Figure S11, Supporting Information). In summary, SP@TIIAn effectively addresses the pharmacokinetic challenges associated with IBS‐D by maintaining stable drug distribution, demonstrating its potential for enhanced drug delivery in IBS‐D patients.

### 2.4 Protective Effects on Gut Epithelial Barrier

**Figure 4:**
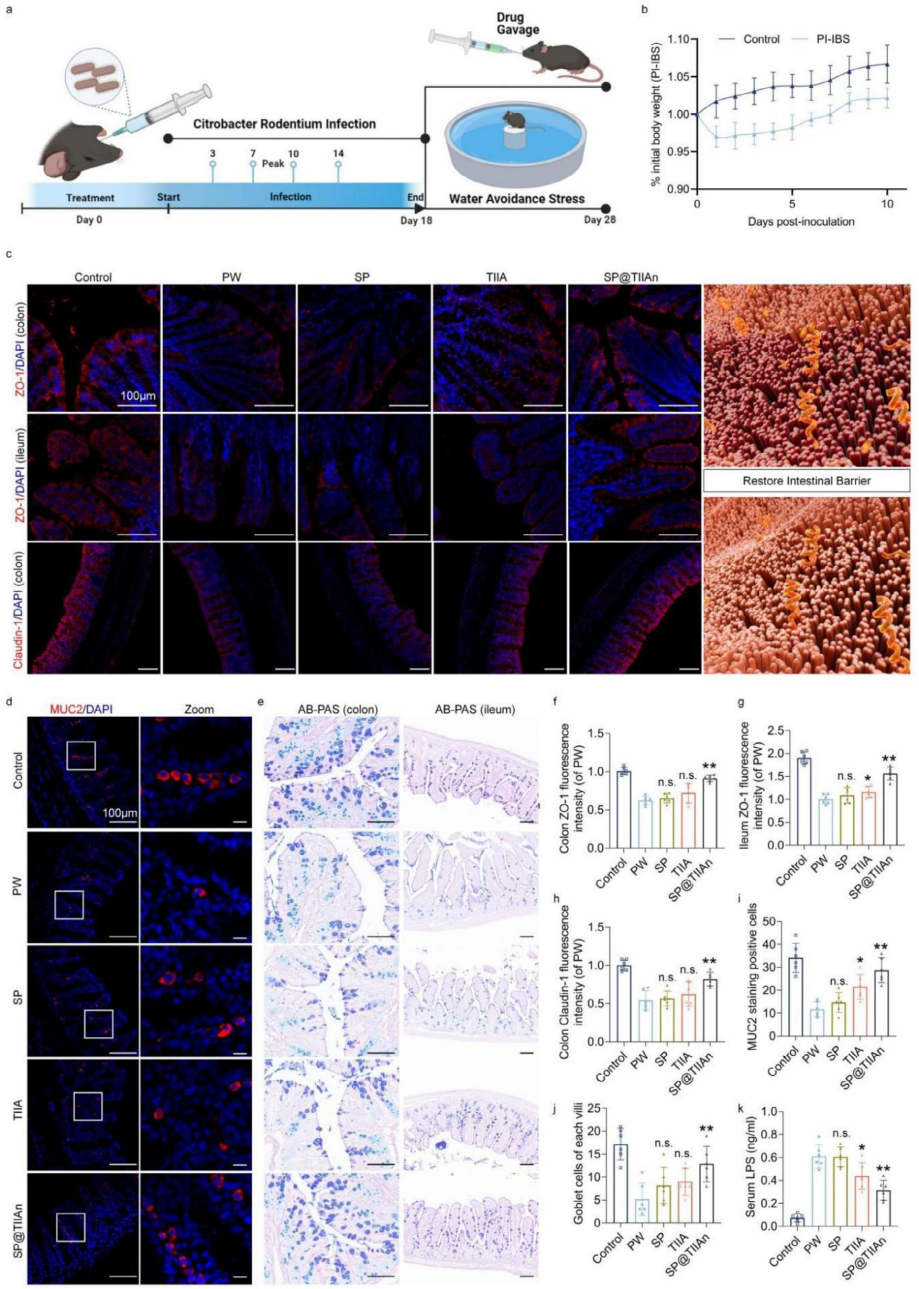
Effects of SP@TIIAn against intestinal epithelial barrier dysfunction. a) Schematic illustration of PW model building and intervention. Created in BioRender. Ji, C. (2025) https://BioRender.com/aws4y45. b) Body weight changes in infected mice. c) Immunofluorescence staining of colon Claudin‐1, ileum ZO‐1, colon ZO‐1. d) Immunofluorescence staining of MUC‐2. e) AB‐PAS staining of intestine. f–i) Fluorescence intensity of colon ZO‐1 (f), ileum ZO‐1 (g), colon Claudin‐1 (h), and MUC‐2 (i). j) The density of goblet cells. k) Serum LPS level. Data are represented as the mean ± SD (*n* = 6). ^*^ represents *p* < 0.05, ^**^ represents *p* < 0.01 versus the PW group, n.s. represents no significance versus the PW group.

Given the multifactorial nature of IBS‐D and its common triggers, such as infection and psychological stress, we utilized Citrobacter rodentium (CR) to induce post‐infectious IBS‐D (PI‐IBS) in mice.^[^ ^27^ ^]^ Following the natural resolution of the infection, we applied the water avoidance stress (WAS) paradigm to simulate psychological stress, thereby further mimicking IBS‐D (PW).^[^ ^28^ ^]^ Fecal CR quantification confirmed complete clearance of the infection by day 18. During this period, the mice exhibited significant weight loss and dehydration due to diarrhea, with a marked difference in weight gain between the PI‐IBS and control groups. Nevertheless, this phenomenon was not witnessed in the subsequent WAS process (**Figure** **4**a,b; Figures S12 and S13, Supporting Information). In IBS‐D, the intestinal mucus barrier and tight junction are frequently compromised, leading to various symptoms such as enhanced pathogen susceptibility and dysbiosis of the gut microbiota.^[^ ^29^ ^]^ Claudin‐1, occludin, and ZO‐1 are key tight junction proteins in the intestinal epithelium, essential for maintaining the integrity of the epithelial barrier.^[^ ^30^ ^]^ The expression levels of both Claudin‐1 and ZO‐1 were significantly reduced in the PW group, while occludin levels remained stable. Treatment with SP@TIIAn effectively restored the intestinal barrier function, whereas the TIIA group exhibited weaker repair, and this is predominantly manifested on ZO‐1 and Claudin‐1 (Figure 4c,f–h; Figure S14, Supporting Information). Furthermore, we examined MUC2, a critical mucin secreted by goblet cells to form a protective mucus barrier.^[^ ^31^ ^]^ In the PW group, a significant reduction in MUC2‐positive goblet cells was observed, accompanied by a decline in mucus barrier integrity. The results demonstrated that SP@TIIAn successfully reduced the decrease in MUC2 protein levels in the colon, further highlighting its potential for protecting the intestinal barrier (Figure 4d,i; Figure S15, Supporting Information). H&E and AB‐PAS staining revealed that the colon of the PW group presented mild inflammatory alterations and a thinned mucus layer, yet no obvious inflammation emerged in the small intestine (Figure 4e,j; Figures S16 and S17, Supporting Information). In addition, impairment of barrier functions resulted in increased permeability of DiO‐Dextran and elevated levels of serum lipopolysaccharide (LPS). These adverse effects can be effectively mitigated by oral administration of SP@TIIAn (Figure 4k; Figure S18, Supporting Information).

### 2.5 Immunomodulation of the Intestinal Microenvironment

**Figure 5:**
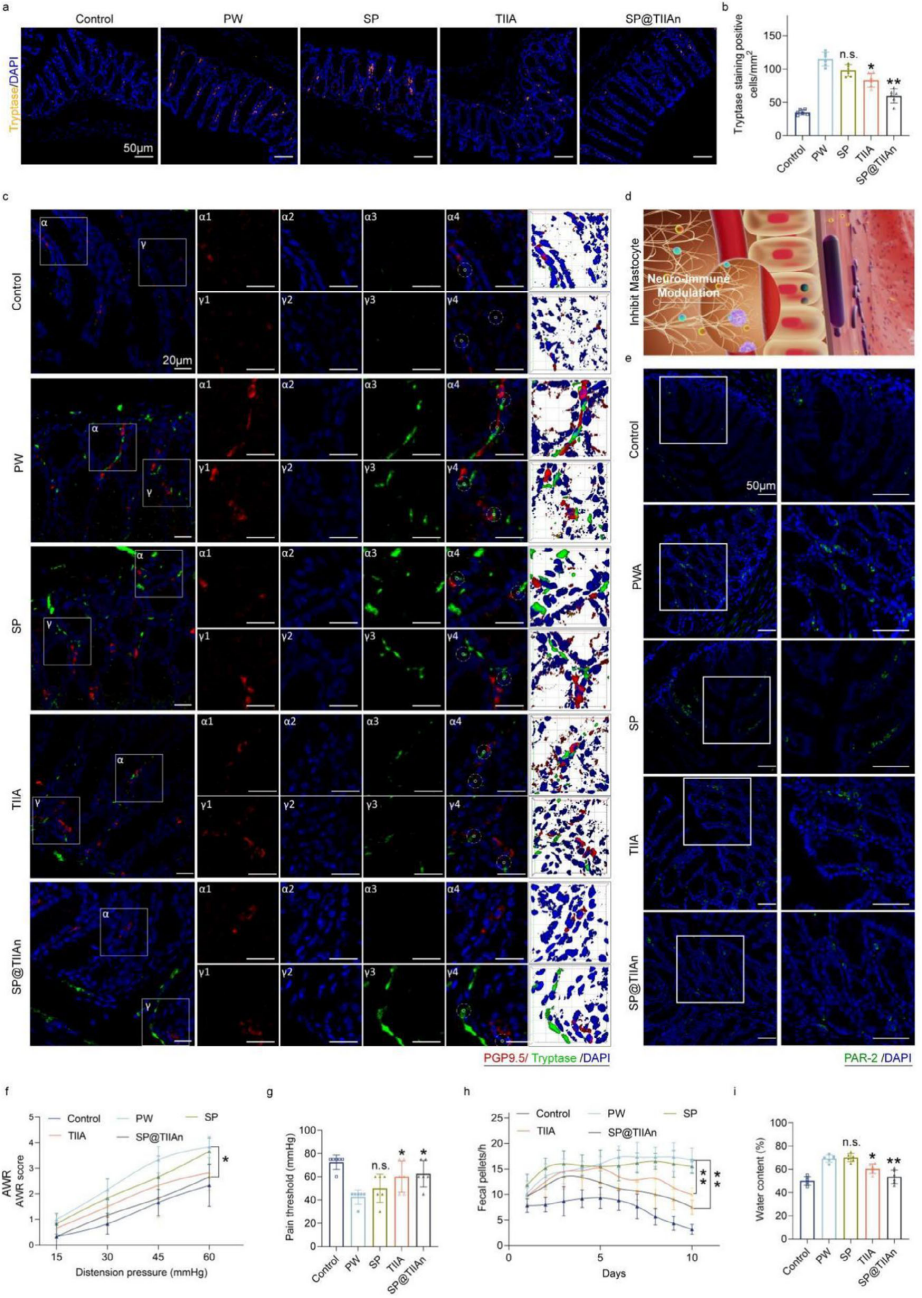
SP@TIIAn suppresses visceral sensitivity and inhibits mast cell activation. a) Immunofluorescence staining of Tryptase and b) density of Tryptase. c) Immunofluorescence staining of Tryptase (red), PGP9.5 (green). d) Schematic diagram of the chemotaxis of mast cells toward nerve fibers. e) Immunofluorescence staining of PAR‐2. f) AWR scores under different pressures. g) The colorectal pressure when reaching the pain threshold. h) the frequency of bowel movements subjected to 1 h of daily WAS. i) Fecal water content of mice. Data are represented as the mean ± SD (*n* = 6). ^*^ represents *p* < 0.05, ^**^ represents *p* < 0.01 versus the PW group, n.s. represents no significance versus the PW group.

The interaction between the enteric nervous system (ENS) and intestinal immune cells is believed to play a critical role in the visceral hypersensitivity and motor alterations observed in IBS‐D.^[^ ^32^ ^]^ Mast cells have garnered particular attention due to their involvement in both IBS‐D and allergic responses.^[^ ^33^ ^]^ In the PW group, we observed a significant increase in the number of colonic mast cells through tryptase staining, which was effectively alleviated by treatment with SP@TIIAn (**Figure** **5**a,b). Subsequently, we focused on regions with a high density of mast cells in each experimental group to investigate their interaction with intestinal nerve fibers (PGP9.5). Through co‐staining with PGP9.5, we observed that activated mast cells were enriched within a 5 µm radius of intestinal nerve fibers, indicating chemotactic enrichment of immune cells and neuro‐immune interactions, which may contribute to the abdominal pain commonly observed in IBS‐D (Figure 5c,d; Figures S19 and S20, Supporting Information). Additionally, protease‐activated receptor‐2 (PAR‐2) exhibited an expression profile similar to that of trypsin (Figure 5e; Figure S21, Supporting Information). Given that visceral hypersensitivity in IBS‐D patients is often characterized by abdominal pain arising during routine activities, we conducted the abdominal withdrawal reflex (AWR) to evaluate colonic sensitivity in animal models. In the PW group, we observed increased AWR scores and decreased pain thresholds, indicative of visceral hypersensitivity. Treatment with SP@TIIAn significantly reduced mast cell activation, preventing their migration toward nerve fibers and suppressing PAR‐2 receptor activation. Consequently, this led to a marked reduction in visceral sensitivity and a noticeable alleviation of abdominal pain symptoms, whereas the effects of TIIA or SP alone were less pronounced (Figure 5f,g). To evaluate diarrhea symptoms in IBS‐D, we quantified the frequency of bowel movements in animals subjected to 1 h of daily WAS. Treatment with TlIA@NP significantly reduced both the frequency of bowel movements and fecal water content, whereas SP@TIIAn exhibited superior efficacy (Figure 5h,i).

### 2.6 Inhibition of Neurogliocyte and Microglial Cell Activation

**Figure 6:**
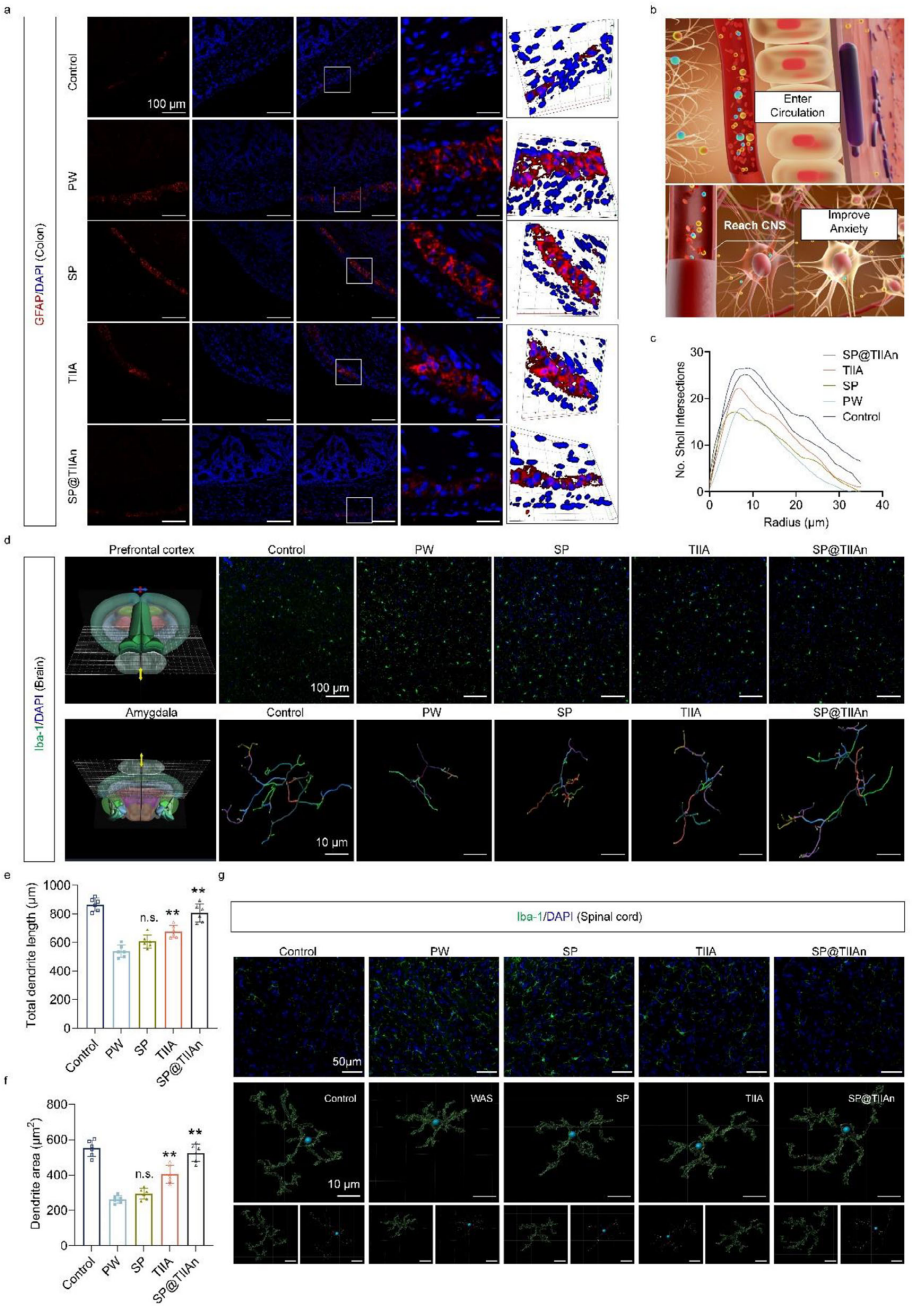
Regulation of neuroglia and microglia by SP@TIIAn. a) Immunofluorescence staining of GFAP. b) Schematic diagram of inhibiting microglial cell activation. c) Sholl analysis of microglia in the amygdaloid nucleus. d) Iba‐1 immunofluorescence staining in the prefrontal cortex and Iba‐1 immunostaining 3D reconstruction in the amygdala, and their localization. e) Dendrite length and f) dendrite area in the amygdaloid nucleus. g) Iba‐1 immunofluorescence staining and Iba‐1 immunostaining 3D reconstruction in spinal cord. Data are represented as the mean ± SD (*n* = 6). ^**^ represents *p* < 0.01 versus the PW group, n.s. represents no significance versus the PW group.

The relationship between enteric glial cells (EGCs) and IBS‐D has garnered increasing attention.^[^ ^34^ ^]^ Activation of EGCs results in the production of pro‐inflammatory cytokines and chemokines, leading to increased gut motility, intestinal permeability, and visceral hypersensitivity.^[^ ^35^ ^]^ EGC proliferation was observed in the PW group, and both TIIA and SP@TIIAn exhibited varying degrees of inhibitory effects on this proliferation (**Figure** **6**a). The alterations observed in the peripheral nervous system prompt us to endeavor to explore whether the central nervous system (CNS) can reap benefits from SP@TIIAn treatment, because small‐molecule drugs or metabolites within the intestinal tract might enter the CNS via the blood circulation (Figure 6b).^[^ ^36^ ^]^ We found a significant increase in the number of microglial cells in the prefrontal cortex, a critical region responsible for maintaining cognitive function. This increase likely contributed to cognitive impairment and weakened recognition memory in PW mice. SP@TIIAn effectively alleviated these issues, demonstrating more pronounced effects compared to TIIA. Next, we examined microglial cells in the amygdala. We found that they were activated and displayed significant morphological changes.^[^ ^37^ ^]^ Activated microglial cells exhibited shortened and thickened processes, thereby reducing their coverage area. SP@TIIAn also alleviated these alterations (Figure 6c–f). Moreover, the dorsal horn of the L6‐S1 spinal cord segment has been reported to be related to the perception of intestinal pain.^[^ ^38^ ^]^ SP@TIIAn likewise inhibited the activation of microglia within it (Figure 6g).

### 2.7 Alleviation of Mental Symptoms

**Figure 7:**
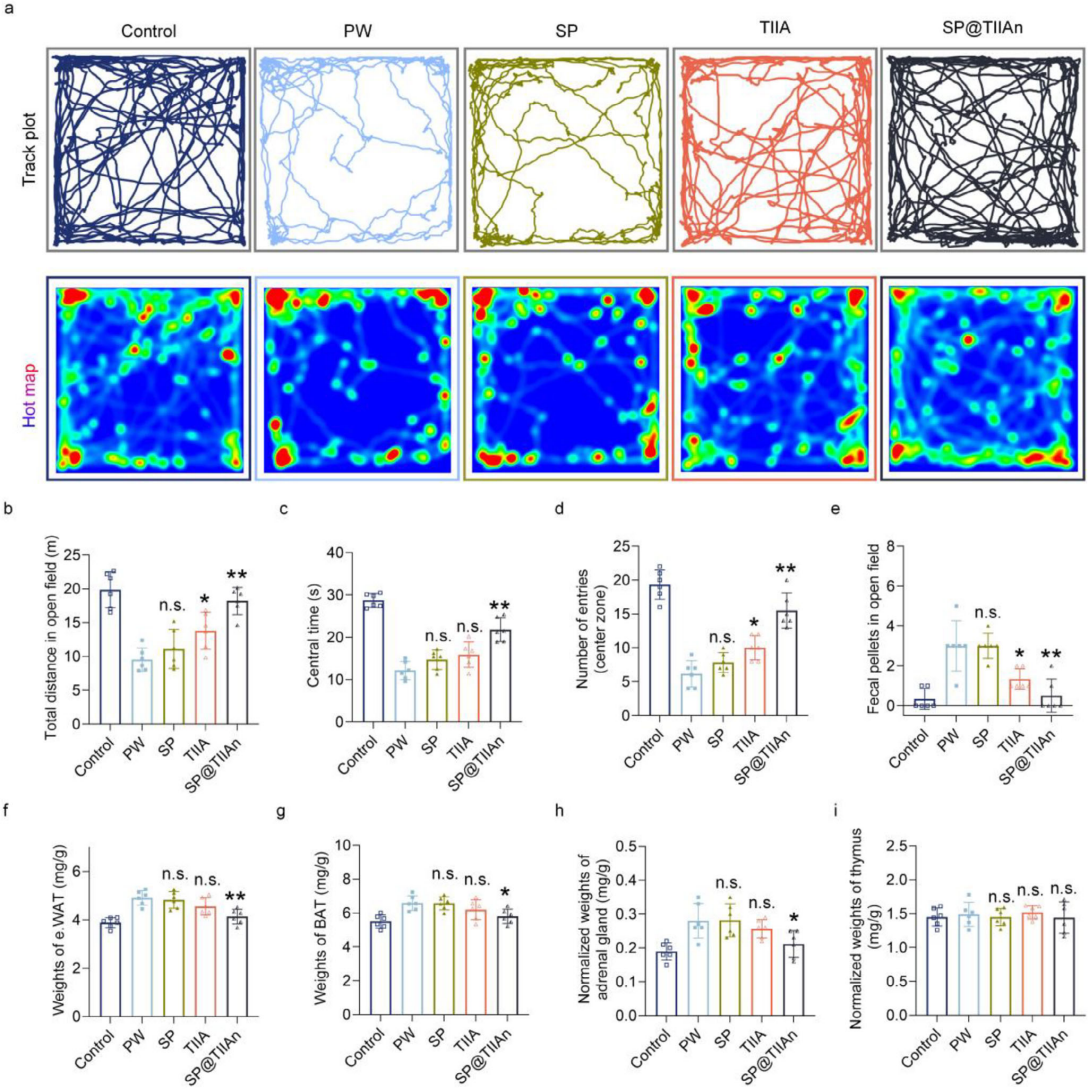
SP@TIIAn prevents behavioral changes in IBS‐D. a) Movement tracking images and heatmaps of mice in the OFT. b) Total movement distance, c) residence time in the central grid, d) number of entries to the central grid, and e) number of fecal pellets in the open field. f,g) The weight of e.WAT (f) and BAT (g). h,i) The weight of the adrenal gland (h), thymus (i). Data are represented as the mean ± SD (*n* = 6). ^*^ represents *p* < 0.05, ^**^ represents *p* < 0.01 versus the PW group, n.s. represents no significance versus the PW group.

Individuals with IBS‐D frequently experience psychological symptoms.^[^ ^39^ ^]^ Therefore, we further investigated the long‐term therapeutic effects of SP@TIIAn on IBS‐D‐associated psychiatric symptoms. In the open field test (OFT), PW mice exhibited reduced movement, shorter duration in the central area, and fewer grid crossings, indicative of anxiety‐like behaviors. Additionally, they showed increased defecation during the OFT, suggesting exacerbated symptoms when exposed to stress in unfamiliar environments. After treatment, SP@TIIAn effectively restored motor function, alleviated anxiety, and improved diarrhea symptoms (**Figure** **7**a–e). Additionally, mice treated with SP@TIIAn showed a marked increase in the time spent exploring and the number of interactions with new objects in the novel object recognition (NOR) test, suggesting enhanced memory and cognitive function (Figure S22a, Supporting Information). To further verify the impact on anxiety, we conducted the elevated plus maze (EPM) test and hole‐board experiments. TIIA exhibited limited and inconsistent efficacy, whereas SP@TIIAn significantly increased the time spent in open arms and the number of crossings in the EPM, as well as the frequency of head dips in the hole‐board test (Figure S22b–e, Supporting Information). Furthermore, it was observed that PW mice exhibited stress‐induced increases in epididymal white adipose tissue (e. WAT) surrounding the testes and brown adipose tissue (BAT) proliferation in the back (Figure 7f,g). Additionally, both adrenal mass and serum corticosterone content were significantly increased due to chronic stress, yet the thymus index remained unaltered. (Figure 7h,i). These findings confirm that IBS‐D can lead to psychiatric‐related symptoms, while administration of SP@TIIAn demonstrated notable alleviating effects.

### 2.8 Regulation of Intestinal Microbiota

**Figure 8:**
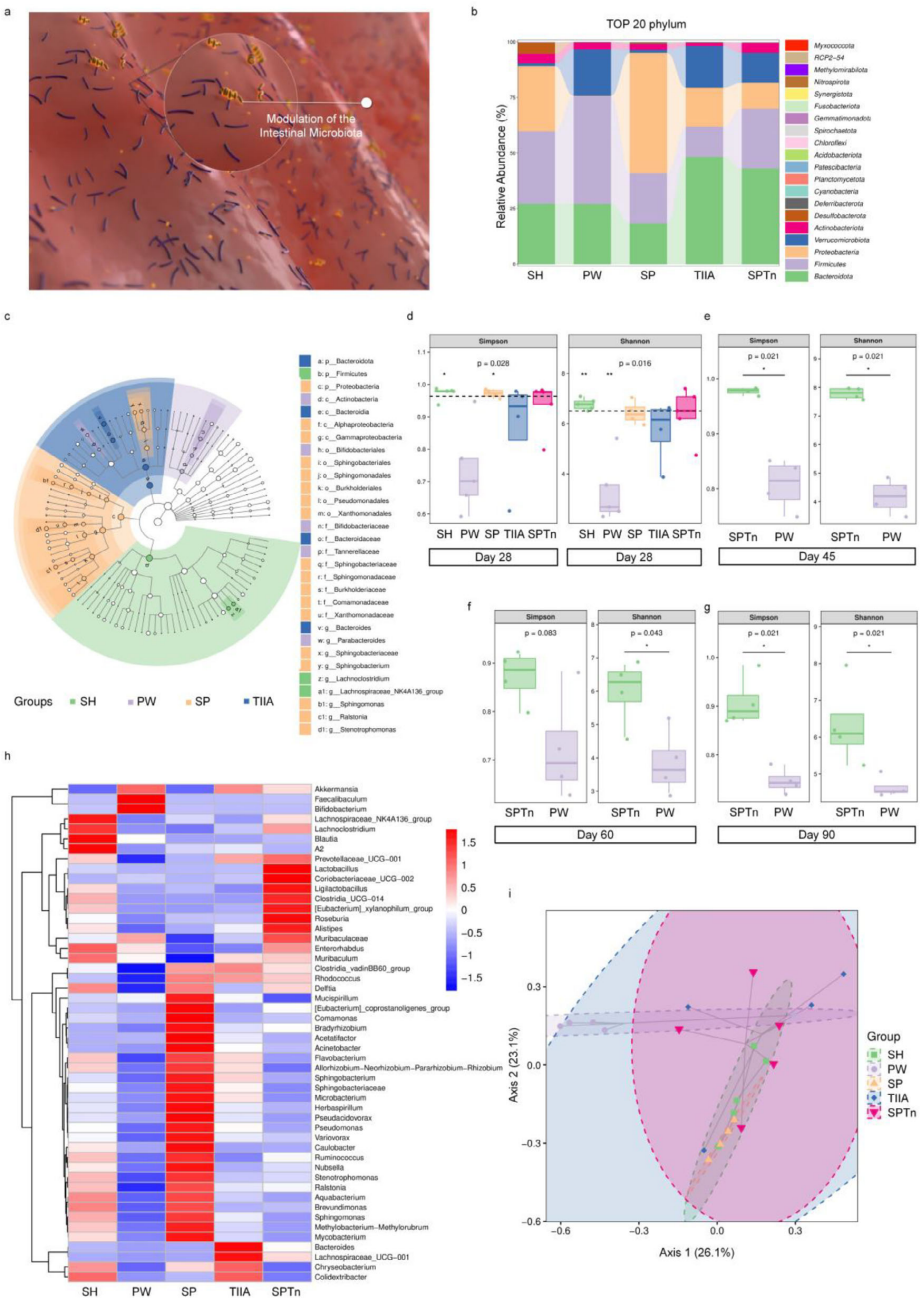
Gut microbiome 16s sequencing. a) Schematic diagram of SP@TIIAn on regulating intestinal microbiota. b) The relative abundance of species composition at the phylum level. c) Classification hierarchy tree‐based group difference classification unit display chart. d–g) Alpha diversity analysis of all groups at different time points. h) heatmap analysis of the relative abundance of microbiota at the genus level. i) Beta diversity analysis for all groups based on PCoA. SH, Control. SPTn, SP@TIIAn. In the SH, PW, and SPTn groups, *n* = 5; while in the SP and TIIA groups, *n* = 4 on the 28th day. At other time points, *n* = 4.

The microbiota has emerged as a crucial player in gut‐brain communication, offering new directions for therapeutic interventions in brain and gastrointestinal diseases.^[^ ^40^ ^]^ 16S amplicon sequencing of stools from each group was conducted to investigate gut microbiota composition (**Figure** **8**a). At the phylum level, an imbalance between Firmicutes and Bacteroidetes was observed in the PW group, indicative of microbiota dysbiosis (Figure 8b,c). α‐diversity analysis, including the Chao1, Shannon, and Simpson indices, revealed a significant reduction in species richness in the PW group, which was effectively restored by SP@TIIAn treatment. Notably, spirulina alone also played a significant role, consistent with its prebiotic properties. During the synthesis process, SP@TIIAn also contains other components. However, we found that this did not cause any adverse effects on the intestinal flora. Moreover, compared with SP and TIIA, SP@TIIAn also fully exerted its function of improving the intestinal flora, which might lead to some improvements in certain phenotypes. (Figure 8d). Over a subsequent extended period, we observed that the SP@TIIAn group continued to exhibit diversity, whereas the PW group did not show signs of recovery (Figure 8e–g). Furthermore, SP@TIIAn significantly enriched the abundance of bacterial genera known to improve IBS‐D symptoms.^[^ ^41^ ^]^ For instance, Roseburia promotes intestinal barrier repair and enhances intestinal barrier function through butyrate production. Collectively, these genera improve gut microecology and restore intestinal health (Figure 8h). No significant differences in β‐diversity were detected among the groups. Nevertheless, the central point of the PW group was relatively distant from those of the other groups (Figure 8i). Furthermore, we measured the butyric acid content in feces. It was found that the content was significantly upregulated in the SP@TIIAn group, and butyrate effectively inhibited microglial activation. These findings suggest that SP@TIIAn might also regulate the gut–brain axis with butyrate as an intermediate, thus alleviating the psychiatric symptoms of IBS (Figure S23, Supporting Information).

### 2.9 Further Evaluation of In Vivo Application

**Figure 9:**
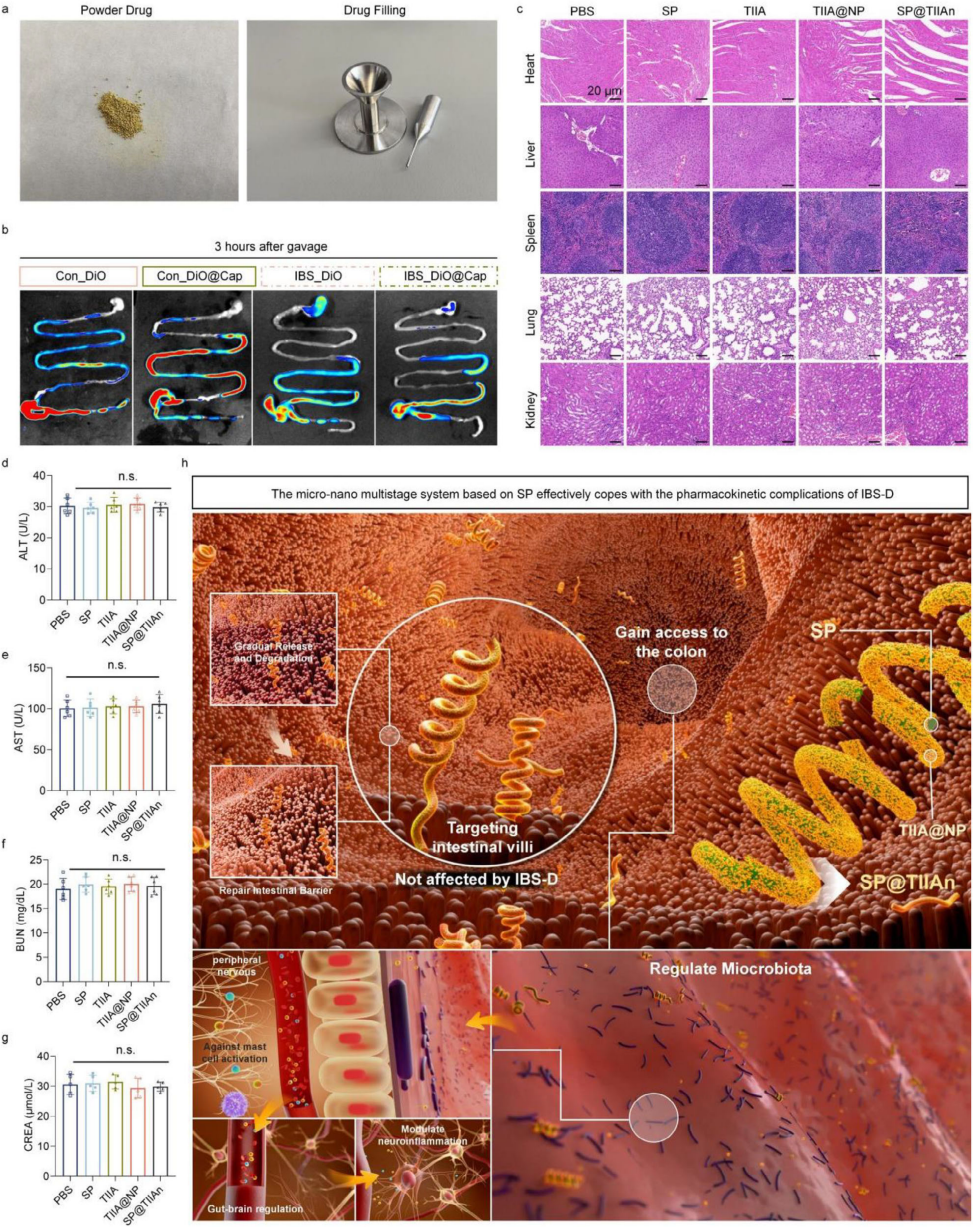
Further exploration of clinical feasibility. a) Preparation of pharmaceutical powders and enteric capsules. b) In vivo distribution of DiO fluorescence in each group. c) Histopathological evaluation of major organs in mice after gavage of different materials. d) Serum ALT, e) AST, f) BUN, and g) CREA. h) A summary schematic diagram of the in vivo therapeutic effect of SP@TIIAn. Data are represented as the mean ± SD (*n* = 6). n.s. represents no significance versus the PW group.

We further validated whether the clinically commonly used enteric‐coated capsules could exert a function analogous to that of SP@TIIAn in the IBS‐D model. For better visualization, DiO was encapsulated within DiO@Cap. The results demonstrated that these capsules effectively provided a sustained‐release effect under normal conditions, but exhibited rapid elimination in the presence of IBS‐D. These discoveries further substantiate the therapeutic superiority of SP@TIIAn under the physiological conditions of IBS‐D and suggest that it is conducive to bridging the clinical void (**Figure** **9**a,b). Finally, due to the known changes in pharmacokinetics, we conducted comprehensive oral biosafety evaluations of SP, TIIA, TlIA@NP, and SP@TIIAn. Following treatment, major organs were excised for histopathological analysis, and serum was collected for biochemical analyses. Both tissue sections and blood biochemical parameters revealed no significant differences between the treatment groups and the control group. In conclusion, no safety concerns were identified, confirming excellent long‐term oral biosafety (Figure 9c–g). Based on the aforementioned results, SP@TIIAn not only effectively copes with the pharmacokinetic complications of IBS‐D that enteric‐coated capsules fail to address, but also exhibits high biological safety (Figure 9h).

### 2.10 In Vitro Experiments of Intestinal Epithelial Cells (IEC‐6) and Microglia (bv2)

Intestinal epithelial cells (IEC‐6) and bv2 microglia were utilized for in vitro experiments. There was no significant impact on cell viability when IEC‐6 cells were incubated with different concentrations of SP, TIIA, TlIA@NP, and SP@TIIAn (Figure S24a, Supporting Information). Moreover, within the applied range of TIIA concentrations, no obvious decline in cell viability was witnessed in bv2 cells (Figure S24b, Supporting Information). Subsequently, we observed that TBHP caused a significant decrease in the levels of tight junction proteins such as ZO‐1 and Claudin‐1, while treatment with TlIA@NP effectively restored their expression. This effect may be attributed to the potential of TIIA to restore intestinal barrier function (Figure S24c,e,f, Supporting Information). Furthermore, by assessing the transepithelial electrical resistance (TEER) of an in vitro IEC‐6 monolayer cell model, we found that both TIIA@NP and SP@TIIAn did not disrupt the barrier (Figure S24g, Supporting Information). Besides, our results demonstrated that LPS stimulation induced microglial cell activation and promoted inflammation, whereas treatment with TIIA effectively reversed this phenomenon, consistent with the anti‐inflammatory properties of TIIA (Figure S24d,h, Supporting Information).

## 3 Conclusion

This study introduces SP@TIIAn, an effective oral drug delivery system combining spirulina and TIIA liposome, as a promising therapeutic approach for IBS‐D. SP@TIIAn effectively targets intestinal villi and enhances intestinal barrier function. It restores the imbalance of the gut microbiota in PW mice by modulating the diversity and composition of the gut microbiome, enriching the abundance of beneficial bacteria. Additionally, SP@TIIAn influences neuroimmune interactions, alleviating psychological symptoms and visceral hypersensitivity. In contrast to the enteric‐coated capsules frequently utilized in clinical settings, SP@TIIAn is conducive to the stable pharmacokinetics of oral medications for IBS‐D patients, a phenomenon that has been scarcely reported in the work of drug delivery system development. Overall, SP@TIIAn offers a comprehensive, multi‐faceted treatment strategy. Nevertheless, there are certain limitations in this study. As an appropriate large animal model for IBS‐D is lacking, more profound verification has not been carried out.

## 4 Experimental Section

### Materials and Reagents

The relevant details can be found in Table S1 (Supporting Information).

### Synthesis and Characterization of TlIA@NP

For the preparation of TIIA@NP, TIIA@LP was initially fabricated via the thin film hydration method. To be specific, a mixture of 100 mg phospholipid, 100 mg cholesterol, and 10 mg TIIA was dissolved in 5 ml chloroform and evaporated at 37 °C and 100 rpm to form a thin film. This film was hydrated with 10 mL of ddH_2_O at 40 °C and 150 rpm. The resultant liposome dispersion was subjected to sonication in an ice bath and then stirred with 5 mg CS at room temperature for 4 h. After centrifugation at 8000 rpm for 8 min, TIIA‐NPs were made to pass through a polycarbonate membrane with a pore size of 100 nm using an extruder (Avanti, Alabama).

### Synthesis and Characterization of SP@TIIAn

Phosphate‐buffered saline (PBS) was used to wash SP. Subsequently, 5 mg of SP was mixed with TlIA@NP (1 mg TIIA) and stirred for 60 min to facilitate interaction. The resulting SP@TIIAn nanoparticles were collected by centrifugation at 5000 rpm for 5 min and washed to remove any unbound TIIA@NP. The surface charges were measured using a dynamic light scattering system (Malvern Panalytical Zetasizer Nano ZS90, UK). UV–vis–near infrared spectra were obtained using a spectrometer (Shimadzu UV‐2600, Japan). The morphological features of the nanoparticles were assessed via TEM (FEI Tecnai F20, USA), SEM (Hitachi SU‐70, Japan), and an upright fluorescence microscope (Nikon, Japan). Additionally, 3D scanning and nano‐coating thickness calculations were performed using a laser confocal microscope (Zeiss LSM880, Germany).

### Simulation of Intestinal Mucus Layer Adhesion In Vitro

To mimic the intestinal mucus layer, mucin was applied to the surface of Au crystals via spin coating at 3000 rpm for 20 s. Equal amounts of TIIA‐LP and TlIA@NP were subsequently introduced into the chamber at the same rate, and the frequency shift (ΔF) was measured in real‐time using a QCM (Biolin QSense Explorer, Sweden).

### Experiments Involving Animals and Cells

All animal procedures were approved by the Institutional Animal Care and Use Committee of Zhejiang University (AIRB‐2021‐952) and were carried out in accordance with the National Institutes of Health Guide for the Care and Use of Laboratory Animals. Female 8‐week‐old Balb/c nude mice and male 5‐week‐old C57BL/6J mice were purchased from Weitonglihua Biotechnology (Beijing, China) and maintained under SPF conditions. Other detailed information can be found in the Supporting Information.

### Inverted Small Intestinal Sac

Equal‐length sections from the same segment of the small intestine were prepared and inverted to expose the villi. These sections were then coated with a uniform layer of DiO‐labeled material in equal amounts. To simulate the action of intestinal fluid, the sections were incubated in a shaker incubator at 37 °C. Finally, fluorescence images were captured using a PhotonIMAGER to assess the distribution of the materials.

### Immunofluorescence

Mouse tissues were collected in accordance with approved ethical guidelines. Mice were anesthetized with isoflurane, and heart perfusion was performed using PBS, followed by 4% paraformaldehyde (PFA) to preserve the integrity of nerve cells and intestinal villi morphology. Tissues were fixed in 4% PFA at 4 °C for 24 h. Paraffin embedding and cryoembedding were conducted according to standard protocols. For fluorescence staining, tissue sections were blocked with 5% bovine serum albumin (BSA) for 30 min and incubated with primary antibodies overnight at 4 °C. After washing, the sections were incubated with the appropriate fluorescent secondary antibodies at room temperature for 2 h, followed by DAPI staining to label cell nuclei. For paraffin sections, antigen retrieval was performed using either sodium citrate buffer or EDTA buffer, depending on the antibody characteristics.

### The Operation Procedures of Various Kits

Detailed experimental procedures and instructions for the various reagents were available in the official product documentation. Refer to Table S1 (Supporting Information) for brand names and product numbers.

### 3D Reconstruction of Microglia, SP@TIIAn, Tryptase and PGP9.5

For 3D reconstruction, images were captured along the *Z*‐axis with a step size of 1 µm and a resolution of 1024 × 1024 pixels. The images were subsequently analyzed using IMARIS software (Bitplane) to quantify dendrite area and length. Sholl analysis was performed using Imaris 9.7 with the Sholl Analysis plugin. Concentric circles were drawn at 1 µm intervals from the cell body, and the number of intersections with microglial processes was quantified. The resulting data were used to evaluate branch complexity and dendritic patterns.

### Statistical Analysis

Data were presented as the mean ± standard deviation (SD). Two‐sided Student's *t*‐tests were employed to compare data between two groups. One‐way ANOVA followed by Tukey's post hoc analysis was used for comparisons involving more than two groups. For the analysis of microbiota alpha diversity, the Wilcoxon test was used to compare differences between two groups, while the Kruskal‐Wallis test was applied for comparisons among multiple groups. Statistically significant P values were indicated in the Figure and legends as ^*^ *p* < 0.05.

## Conflict of Interest

The authors declare no conflict of interest.

## Supporting information



Supporting Information

Supporting Information

## Data Availability

The data that support the findings of this study are available from the corresponding author upon reasonable request.
