# Supplementary material for: An Effective Oral Drug Delivery Route for Pharmacokinetic Complications: Spirulina Lipid Nanotechnology System
Source: Adv Sci (Weinh). 2025 Sep 12;12(44):e09731. doi: 10.1002/advs.202509731 (PMC12667466; doi:10.1002/advs.202509731)
Supplement: Supplementary file 1 — Supporting Information [file ADVS-12-e09731-s001.docx]

Supporting Information

**An Effective Oral Drug Delivery Route for pharmacokinetic complications: Spirulina Lipid Nanotechnology System**

Jiahui Ye#, Xinyi Wu#, Jin Liu#, Yongmi Guo, Chong Ji, Zhouyue Wang, Di Yang, Min Zhou*

* The corresponding authors

[zhoum@zju.edu.cn](mailto:zhoum@zju.edu.cn) (M.Z.)

**Experimental Section**

**Table S1. REAGENT or RESOURCE**

| REAGENT or RESOURCE | SOURCE | IDENTIFIER |
| --- | --- | --- |
| Antibodies | | |
| Mucin 2/MUC2 | Santa Cruz Biotechnology | Cat# sc-7314; RRID: AB_627970 |
| ZO-1 | Abcam | Cat# ab221547; RRID: AB_2892660 |
| Occludin | Abcam | Cat# ab216327; RRID: AB_2737295 |
| Claudin-1 | ThermoFisher | Cat# 71-7800; RRID: AB_AB_2533997 |
| Iba-1 | Abcam | Cat# ab178846; RRID: AB_2636859 |
| Mast Cell Tryptase | Santa Cruz Biotechnology | Cat# [sc-59587; RRID:AB_793510](https://www.scbt.com/zh/p/mast-cell-tryptase-antibody-aa1) |
| UCH-L1/PGP9.5 | Protein tech | Cat# [14730-1-AP; RRID:AB_2](https://www.scbt.com/zh/p/mast-cell-tryptase-antibody-aa1)210497 |
| PAR-2/SAM11 | Santa Cruz Biotechnology | Cat# sc-13504; RRID: AB_628101 |
| GFAP | Abcam | Cat# ab7260; RRID: AB_305808_ |
| *Salmonella* | Abcam | Cat# ab35156; RRID: AB_777811 |
| CD86/B7-2 | Santa Cruz Biotechnology | Cat# sc-28347; AB_627200 |
| Goat Anti-Rabbit IgG H&L (Alexa Fluor 488) preadsorbed | Abcam | Cat# ab150081; RRID: AB_2734747 |
| Goat Anti-Mouse IgG H&L (Alexa Fluor 555) preadsorbed | Abcam | Cat# ab150118; RRID: AB_2714033 |
| Goat Anti-Mouse IgG H&L (Alexa Fluor 647) preadsorbed | Abcam | Cat# ab150119; RRID: AB_2811129 |
| Bacteria, microalgae and cell lines | | |
| *S. platensis* | Guangyu Biological Technology | Cat# GY-D18 |
| *Citrobacter rodentium* | MingZhou Biotechnology | Cat# BMZ151459, ATCC 51459 |
| *Salmonella Enterica Typhimurium SL1344* | Biobw Biotechnology | Cat# Bio-110527 |
| Bv2 | Cellverse Bioscience Technology | Cat# iCell-m011 |
| IEC-6 | Cellverse Bioscience Technology | Cat# iCell-r016 |
| Chemicals, peptides, and recombinant proteins | | |
| Tanshinone IIA | Aladdin | Cat# T109795 |
| Cholesterol | Aladdin | Cat# C104032 |
| Lecithin | Aladdin | Cat# L105734 |
| FITC-Dextran 4kDa | Sigma-Aldrich | Cat# 60842-46-8 |
| Ammonium chitosan | Macklin | Cat# 850126 |
| 3,3'-Dioctadecyloxacarbocyanine Perchlorate (DiO) | YEASEN | Cat# 40725ES25 |
| Mucin | Beijing Solarbio Science & Technology | Cat# M7630 |
| Indian ink | Shanghai Yuanye Biotechnology | Cat# S30881 |
| Acetonitrile | Sinopharm Chemical Reagent | Cat# [400641646](https://www.reagent.com.cn/goodsDetail/Acetonitrile/%E4%B9%99%E8%85%88/f6f5f223116646b5aed59e709a14eba4) |
| Maconkey Agar | Beijing Solarbio Science & Technology | Cat# M8560 |
| DAPI Fluoromount-G | Southern Biotechnology | Cat# 0100-20 |
| Hoechst 33342 | Beyotime Biotechnology | Cat# C1026 |
| Sodium Citrate buffer | Beijing Solarbio Science & Technology | Cat# C1010 |
| Sodium butyrate | Aladdin | Cat# S102954 |
| Tris-EDTA | Beijing Solarbio Science & Technology | Cat# C1038 |
| Quick Antigen Retrieval Solution for Frozen Section | Beyotime Biotechnology | Cat# P0090 |
| Tween-20 | Beijing Solarbio Science & Technology | Cat# T8220 |
| Tissue-Tek O.C.T. Compound | SAKURA | Cat# 4583 |
| BSA, Fraction V | Beyotime Biotechnology | Cat# ST023 |
| Triton X-100 | Beijing Solarbio Science & Technology | Cat# T8200 |
| Fetal Bovine Serum | Gibco | Cat# 10099141C |
| Critical commercial assays | | |
| Hematoxylin-Eosin(HE) Stain Kit | Beijing Solarbio Science & Technology | Cat# G1120 |
| Alcian Blue Periodic Acid Schiff(AB-PAS) Stain Kit | Beijing Solarbio Science & Technology | Cat# G1285 |
| ELISA Kit for Lipopolysaccharide (LPS) | USCN KIT INC. | Cat# CEB526Ge |
| Mouse Corticosterone ELISA Kit | Uping Biotechnology | Cat# YPJ1166 |
| Calcein-AM/PI Double Stain Kit | YEASEN | Cat# 40747ES76 |
| Reactive Oxygen Species Assay Kit | YEASEN | Cat# 50101ES01 |
| Cell Counting Kit (CCK-8) | YEASEN | Cat# 40203ES60 |

**Table S2. AWR scoring**

| Characteristic | Score |
| --- | --- |
| No behavioral response to CRD | 0 |
| A brief head movement, then no movement | 1 |
| The abdominal muscles contract but the abdomen does not lift | 2 |
| The abdomen is raised without pelvis and scrotum | 3 |
| Arch the body and lift the perineum | 4 |

**Animal experiments**

All animal procedures were approved by the Institutional Animal Care and Use Committee of Zhejiang University (AIRB-2021-952) and were carried out in accordance with the National Institutes of Health Guide for the Care and Use of Laboratory Animals. Female 8-week-old Balb/c nude mice and male 5-week-old C57BL/6J mice were purchased from Weitonglihua Biotechnology (Beijing, China) and maintained under SPF conditions.

**In vivo distribution evaluation**

After a 12-hour fasting period, eight-week-old BALB/c Nude mice were orally administered free DiO and various DiO-labeled materials. Fluorescence images of live and ex vivo samples were collected at various time points using the PhotonIMAGER™ (Biospace, USA). Intestinal tissues were frozen-sectioned for observation, and SEM analysis was performed on intestinal contents at specified time points.

**Uptake of TIIA@LP and TIIA@NP by IEC-6**

IEC-6 cells were seeded into 24-well plates at a density of 10,000 cells per well and co-incubated with DiO-labeled TIIA-LP or TlIA@NP 24h later. The fluorescence intensity of the IEC-6 cell membrane was measured at various time points to assess cell uptake efficiency.

**Preparation of samples for SEM**

The samples were first fixed in a 2.5% glutaraldehyde solution at 4°C overnight, followed by three washes with PBS (15 minutes each). They were then post-fixed in 1% osmic acid for 1–2 hours and washed three additional times with PBS. After fixation, the samples underwent a graded ethanol dehydration process and were further dehydrated using a critical point dryer (Hitachi HCP-2, Japan). Prior to imaging, ion sputtering was performed for 5 minutes using an ion sputtering coater (Hitachi E-1010, Japan) to prepare the samples for observation

**Salmonella typhimurium infection experiment**

Salmonella typhimurium was used to assess the therapeutic efficacy in treating SIBO. To facilitate bacterial colonization, mice were pretreated with an intragastric dose of streptomycin (1 g/kg) 24 hours prior to infection. Starting on the second day, different therapeutic interventions were administered once daily for four consecutive days. Intestinal tissues were subsequently collected for immunofluorescence staining to evaluate the effects of the interventions.

Grouping Information:

1. Salmonella + PBS;

2. Salmonella + SP ((250 mg/kg));

3. Salmonella + TIIA (50 mg/kg);

4. Salmonella + TIIA@NP (50 mg TIIA/kg)

5. Salmonella + SP@TIIAn (50 mg TIIA/kg).

**Ink propulsion experiment**

To induce symptoms similar to those of IBS-D, mice were administered an intragastric dose of 300 μL of castor oil. After symptom onset, they received an intragastric administration of 200 μL of India Ink. Following either a 15-minute or 30-minute period, the entire small intestine and colon, from the distal pylorus to the anus, were isolated to measure the distance of ink propulsion. The “Ink part of whole small intestine” refers to the percentage of the farthest distance that ink has advanced in relation to the total length of the small intestine.

**In-vivo distribution and pharmacokinetics in the IBS-D model**

IBS-D mice received intragastric administration of DiO-labeled TlIA@NP and SP@TIIAn. Fluorescence imaging of both in vivo and ex vivo samples was conducted using the PhotonIMAGER™, and intestinal tissues from various regions were analyzed using fluorescence microscopy and SEM. Additionally, blood and intestinal tissue samples were collected for drug concentration measurements, following the specific analytical procedures described in the LC-MS/MS protocol.

**LC-MS/MS**

Mice were fasted for 12 hours before receiving intragastric administration of TlIA@NP and SP@TIIAn. At designated time points post-administration, tissues and blood samples were collected and processed into homogenates and serum, respectively, followed by centrifugation at 10,000 rpm for 10 minutes. To precipitate proteins, 150 μL of acetonitrile was added to 50 μL of intestinal homogenate or serum. After thorough mixing, the samples underwent a second centrifugation under the same conditions. The resulting supernatant was immediately collected and analyzed using an Agilent 1290 series system (Agilent, Waldbronn, Germany).

**IBS-D animal model**

To construct the IBS-D mouse model, the process was divided into two phases: CR infection and subsequent water avoidance stress (WAS). CR was cultured in Luria Broth medium at 37°C. Each mouse received an oral gavage of 2 × 10^9 CFU of CR, while the control group received an equivalent volume of sterile PBS via gavage. Fecal samples were collected post-infection and inoculated onto MacConkey agar to assess the infection status. After confirming complete eradication of the infection, mice were subjected to a daily one-hour WAS protocol over a 10-day period on an elevated platform positioned 1 cm above the water surface. The control group was similarly placed on the platform without water (sham WAS). Treatment (PBS, SP, TIIA, SP@TIIAn) was administered following each daily WAS session. The number of defecations and fecal moisture content were recorded upon completion of the experiment.

**Intestinal permeability in vivo**

Intestinal cell paracellular permeability was evaluated using 4 kDa DiO-Dextran. Mice were gavaged with 200 µL of sterile 1X PBS containing DiO-Dextran (75 mg/mL). Serum samples were collected 5 hours post-gavage, and fluorescence was quantified using the SpectraMax®iD5 (Molecular Devices, USA) with excitation at 485 nm and emission at 522 nm.

**Abdominal withdrawal reflex (AWR)**

After brief isoflurane anesthesia, a pediatric silicone balloon catheter was gently inserted into the rectum, with the balloon positioned 2.5–3 cm from the anal verge, and secured in place. Following a 30-minute recovery period to allow the mice to acclimate, colorectal dilation was initiated using gradual pressure increments. The pressures applied were 15, 30, 45, and 60 mmHg, each maintained for 20 seconds, with a 5-minute interval between applications. The pain threshold was determined when the AWR score reached 3. Details of the AWR scoring system are provided in **Supplementary Table 2**.

**Open field test (OFT)**

The OFT was employed to quantify anxiety-like behaviors. Prior to any behavioral experiments, animals were acclimated to the experimental environment for a 2-hour period. Mice were then positioned in the center of a 45 cm diameter open field, and their movements were documented for 5 minutes using a camera system. The central area of the field was defined, and the following parameters were assessed: total distance traveled, duration spent in each distinct zone, number of shuttle movements, and number of defecations.

**The elevated plus maze (EPM)**

The elevated plus maze consists of two open arms (37 × 5 × 0.6 cm) and two closed arms (37 × 5 × 15 cm), arranged perpendicularly in a cross configuration. The maze was elevated 50 cm above the floor. Mice were placed in the central square, facing an open arm, and their behavior was recorded for 5 minutes. Parameters assessed included the time spent exploring the open arms and the number of shuttle crossings between arms.

**Novel object recognition (NOR) test**

The day before the experiment, individual mice were acclimated to a standardized open field (45 × 45 × 40 cm) for 10 minutes. On the experimental day, mice were placed in the open field with two identical objects arranged symmetrically, and their activity was recorded for 10 minutes. After a one-hour interval in their home cages, one of the objects was replaced with a novel object, and the mice were reintroduced into the open field. The time spent exploring and the number of investigations directed toward the novel object were recorded. Exploration was defined as directing the snout towards and sniffing the object; locomotion or climbing on the object was not considered exploration. The Discrimination Index was calculated as the difference in time spent exploring the novel object versus the familiar object.

**Hole-board experiment**

The hole board apparatus consists of a 40 cm × 40 cm base with 16 holes (3 cm in diameter) evenly distributed across the surface. Mice were placed in the center of the board, and their activity, specifically the number of hole entries, was recorded during a 5-minute period using infrared sensors located within each hole.

**Cell experiments**

Bv2 cells were cultured in high-glucose DMEM, while IEC-6 cells were maintained in high-glucose DMEM supplemented with 0.01 mg/mL bovine insulin. All media were further supplemented with 10% fetal bovine serum (FBS) and 1% penicillin-streptomycin. In the experiments targeting IEC6 cells, the concentration of TBHP was 200 μM, and that of SP@TIIAn was 10 μg TIIA/ml. The other groups employed the equivalent amounts of SP and TIIA. In the experiments concerning bv2 cells, the concentration of LPS was 1 μg/mL, and the concentration of TIIA was 0.5 μg/ml.

**16S rDNA amplicon sequencing**

Fecal microbial DNA was extracted from mouse fecal samples and quantified using a Nanodrop spectrophotometer, and the quality of the extracted DNA was detected by 1.2% agarose gel electrophoresis. The target fragment was amplified by PCR, and the resulting products were purified using magnetic beads and recovered by fluorescence quantification. A sequencing library was subsequently prepared for high-throughput sequencing. Microbial composition and biodiversity were subsequently evaluated, with alpha diversity assessed using the Chao1 index and beta diversity visualized through principal coordinate analysis (PCoA) based on Bray-Curtis distance.

**The pharmacokinetics of DiO and DiO@Cap**

The enteric capsule equipment and raw materials were sourced from Yuyan Medical Instrument Company (Shanghai, China). Detailed usage guidelines are available on their official website (http://www.yuyanbio.com). Both free DiO and capsule formulations (DiO@Cap) were orally administered to IBS-D and control model mice to investigate in vivo distribution.

Grouping Information:

1. Con_DiO: Normal mice received free DiO via gavage.
2. Con_ DiO @Cap: Normal mice received the capsule form of DiO via gavage.
3. IBS_ DiO: IBS-D mice received free DiO via gavage.
4. IBS_ DiO @Cap: IBS-D mice received the capsule form of DiO via gavage.

**Biosafety evaluation**

Male C57BL/6J mice (5 weeks old) were orally administered SP@TIIAn at a dose of 50 mg/kg TIIA once daily for 30 days. The other groups received equivalent doses of SP, TIIA, TlIA@NP, or PBS. Following the treatment period, mice were euthanized for blood collection and tissue dissection. Serum samples were analyzed for liver and kidney function markers. Major tissues, including the heart, liver, spleen, lung, and kidney, were fixed in paraformaldehyde, embedded in paraffin, sectioned, and stained with H&E for histological evaluation.

**
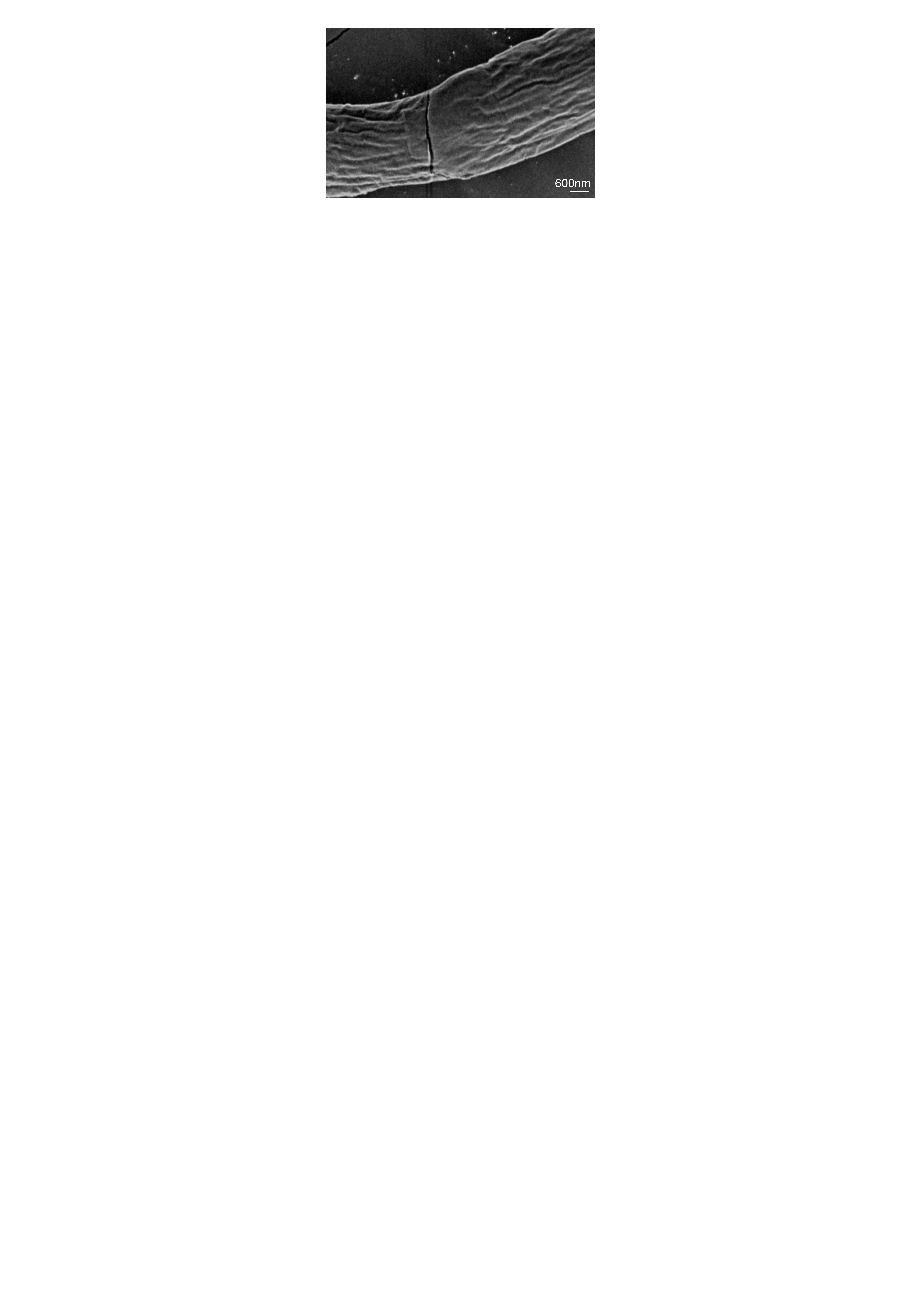
**

**Figure S1.** The original SEM images of SP in Figure 1d.


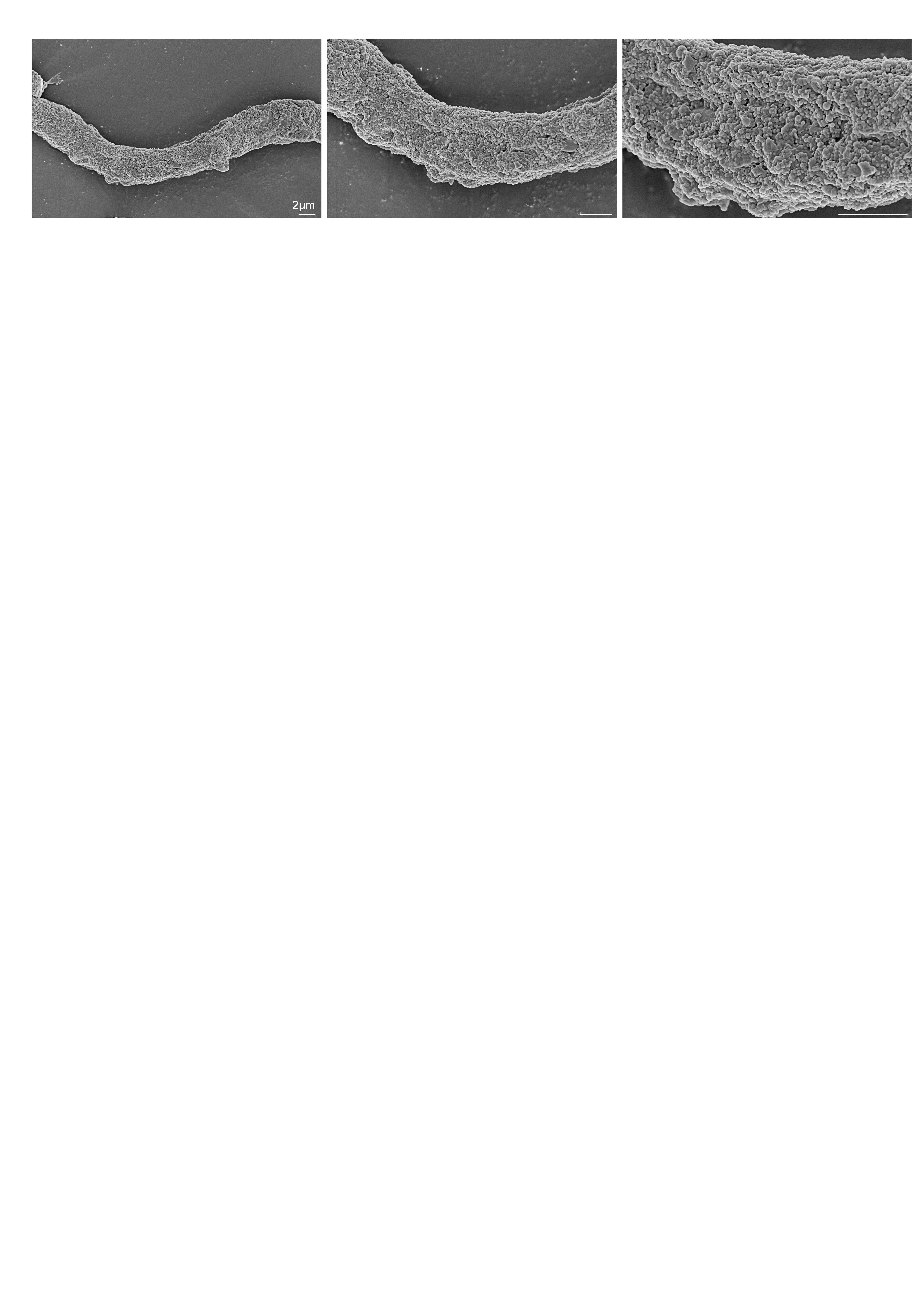


**Figure S2.** The original SEM images of SP@TIIAn in Figure 1k.


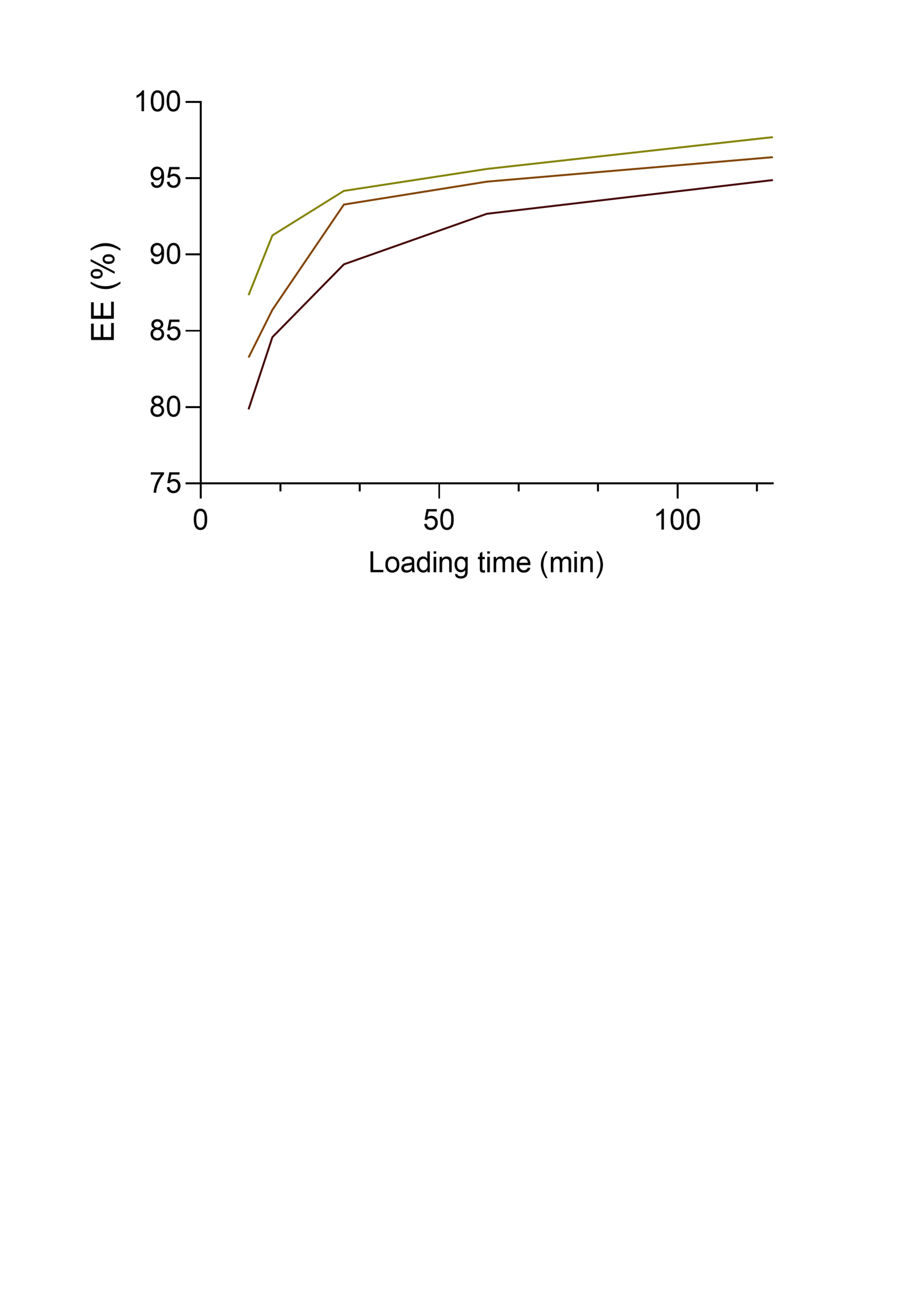


**Figure S3.** EE (%) of SP@TIIAn at different reaction times (n=3).


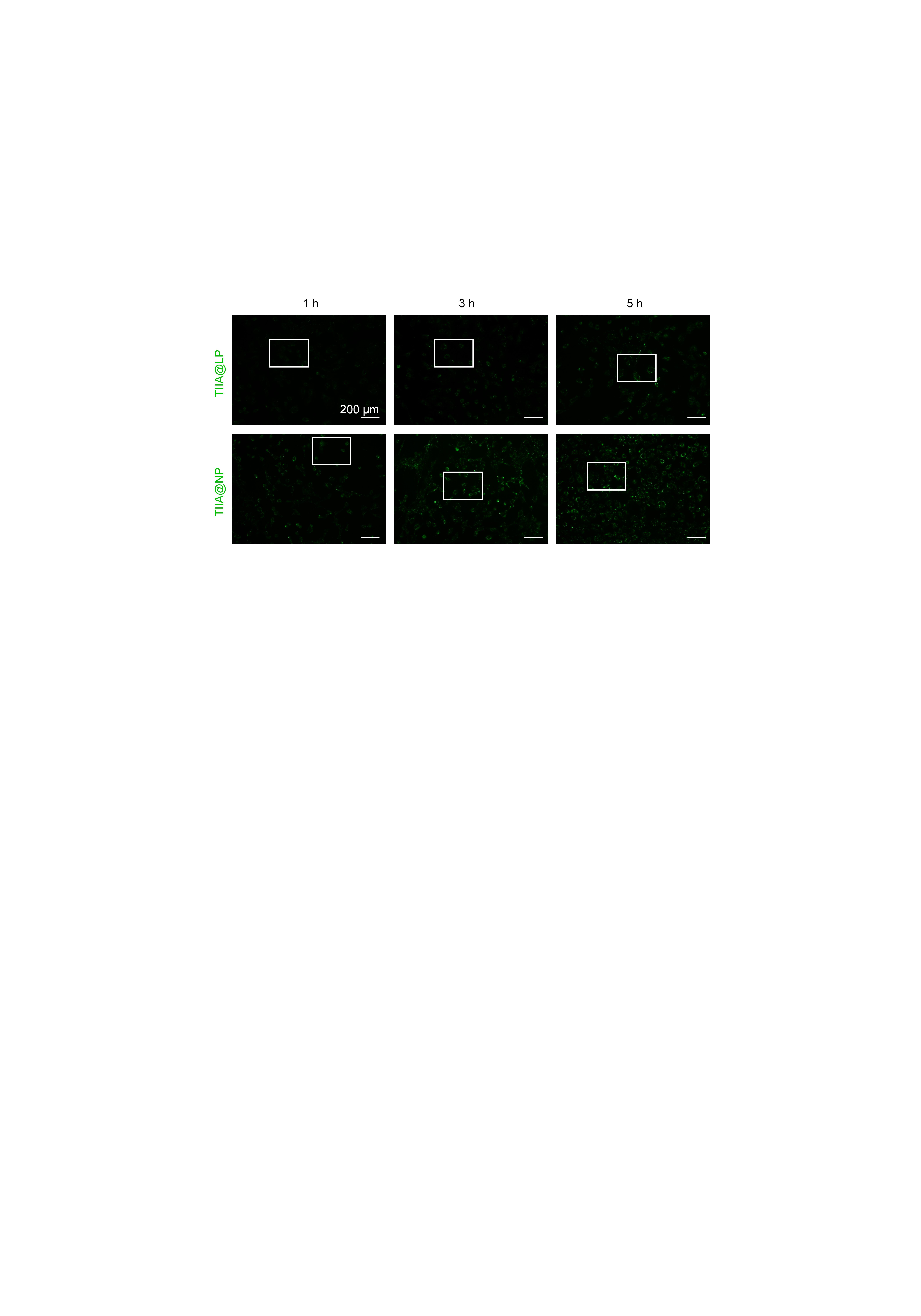


**Figure S4.** larger area of Figure 2a.


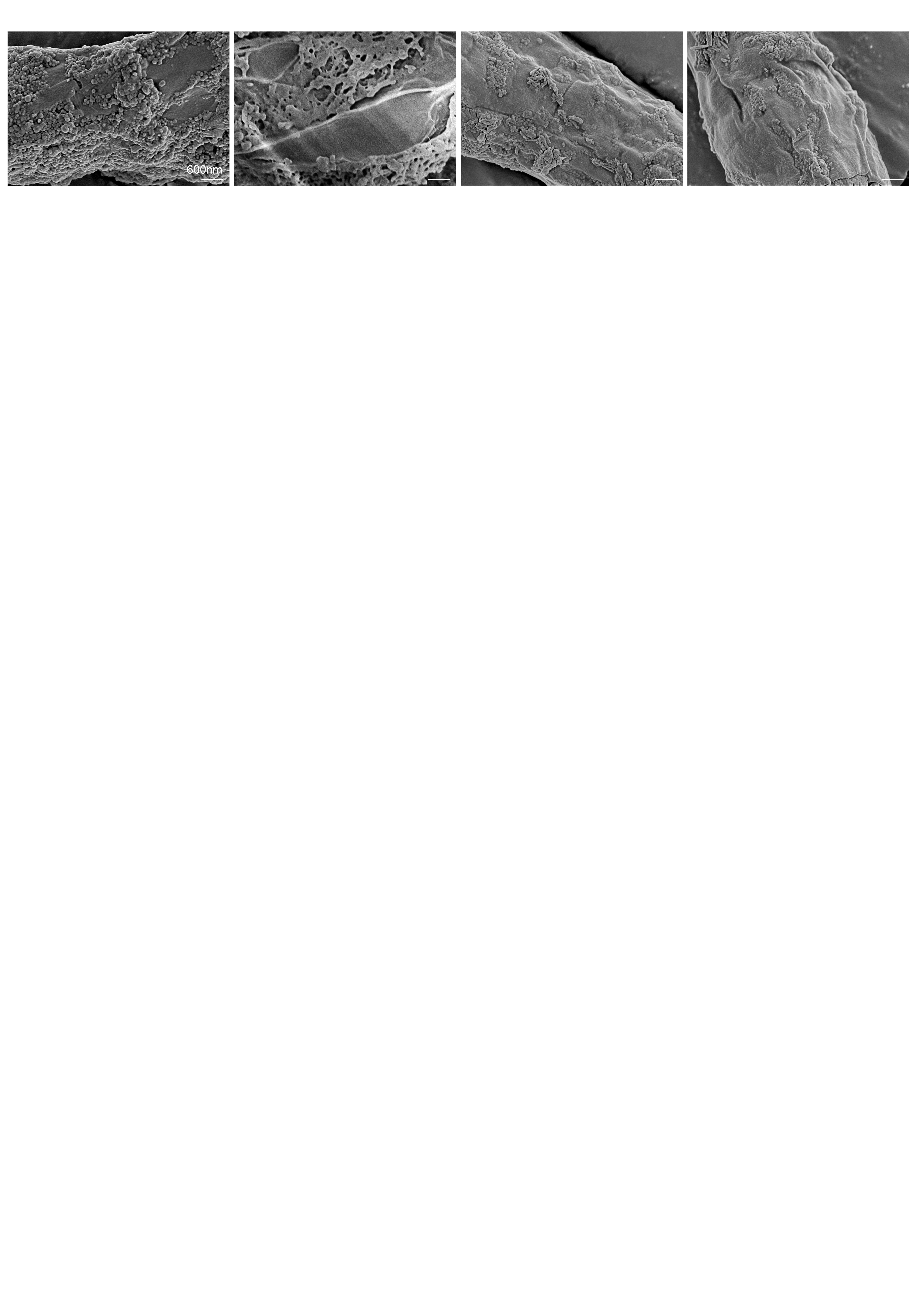


**Figure S5.** The original SEM images of SP@TIIAn in Figure 2d.


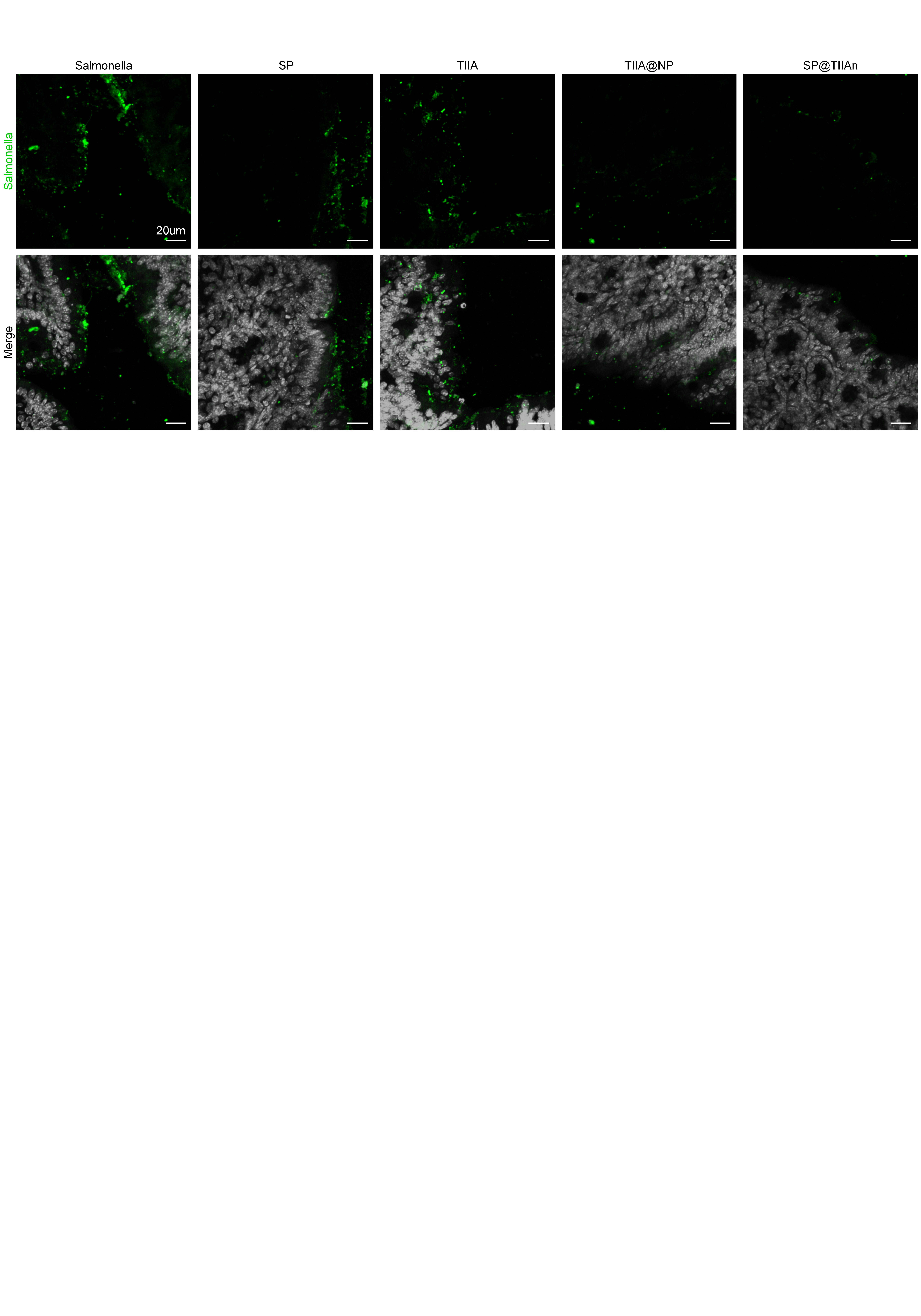


**Figure S6.** 2D images of Figure 2e.


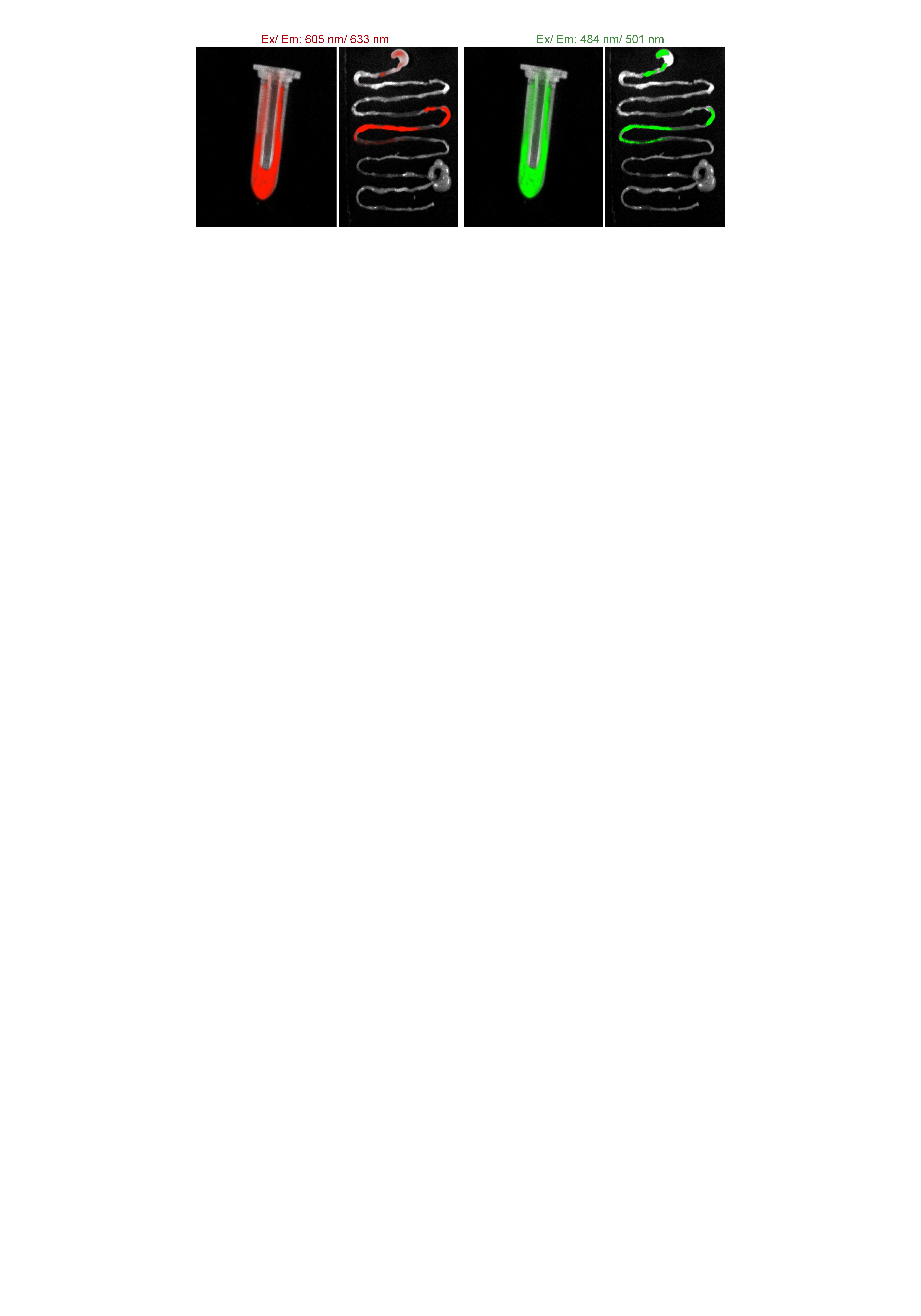


**Figure S7.** Examples of fluorescence images of the SP channel and DiO channel of SP@TIIAn.


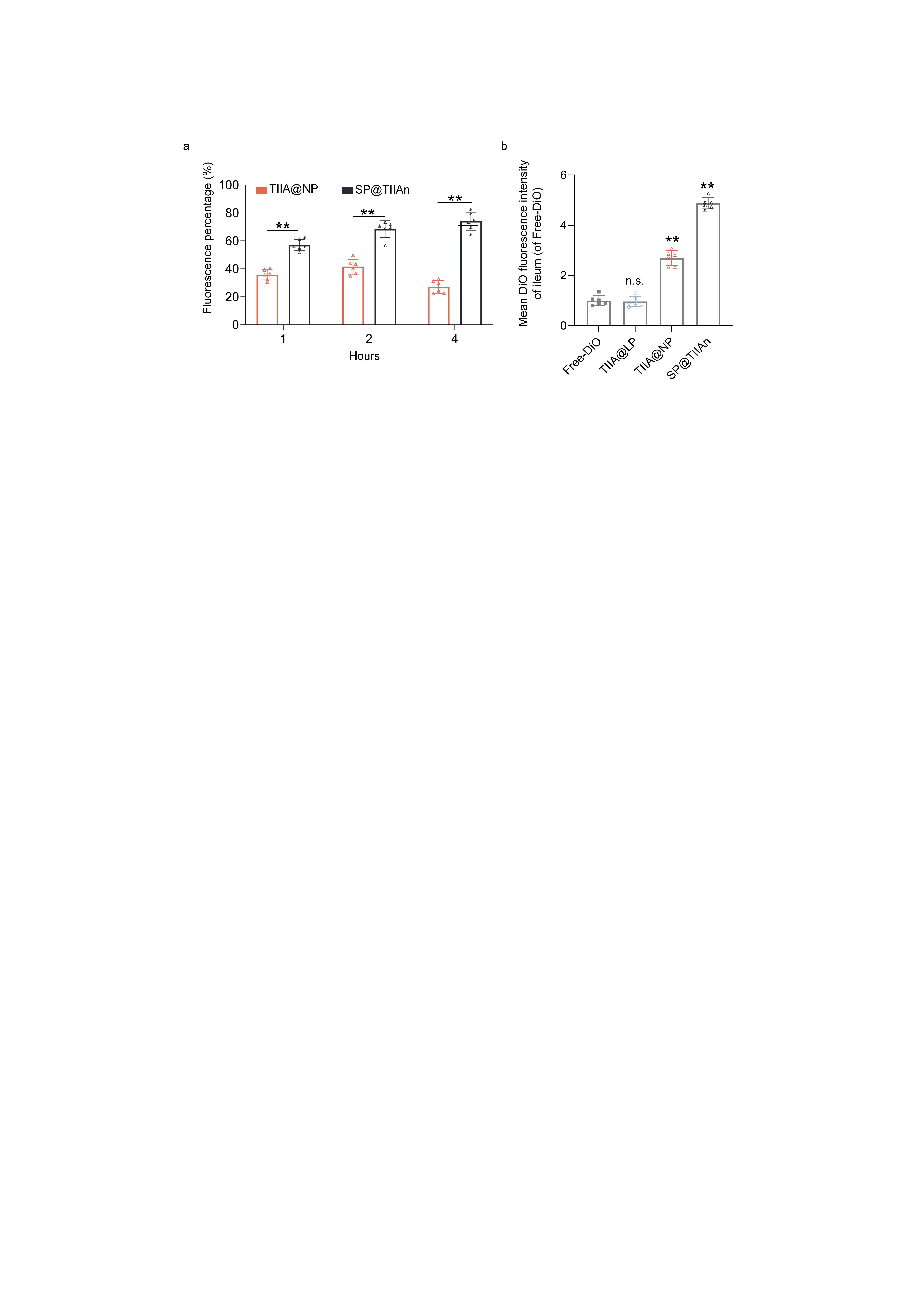


**Figure S8.** a) The proportion of the small intestine area occupied by fluorescence based on an appropriate threshold. b). DiO fluorescence intensity of ileal frozen sections 4 hours after gavage. Data are represented as the mean ± SD (n = 6). ** represents *P* < 0.01 vs. the PW group, n.s. represents no significance vs. the Free-DiO group.


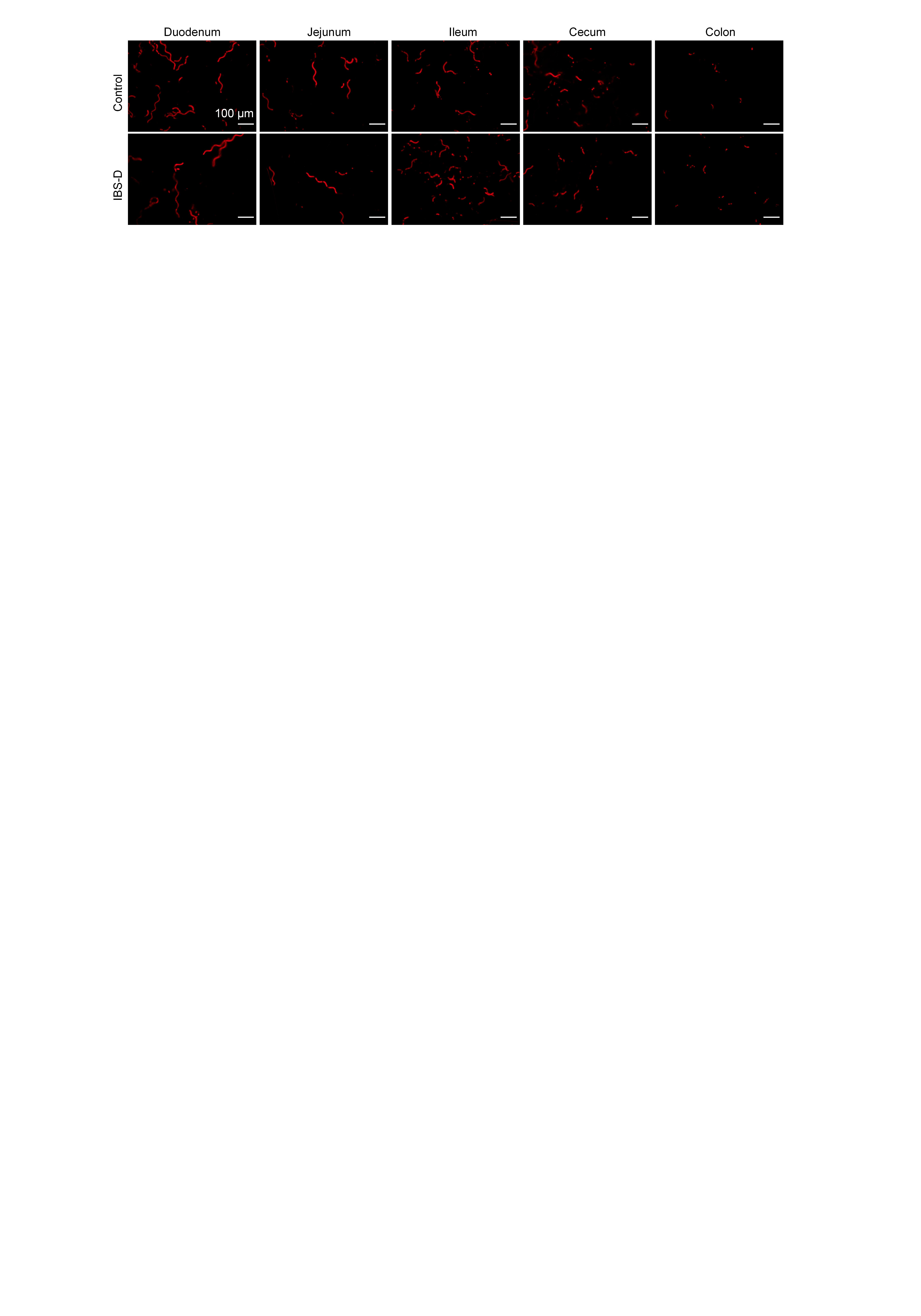


**Figure S9.** SP in intestinal contents of different parts.


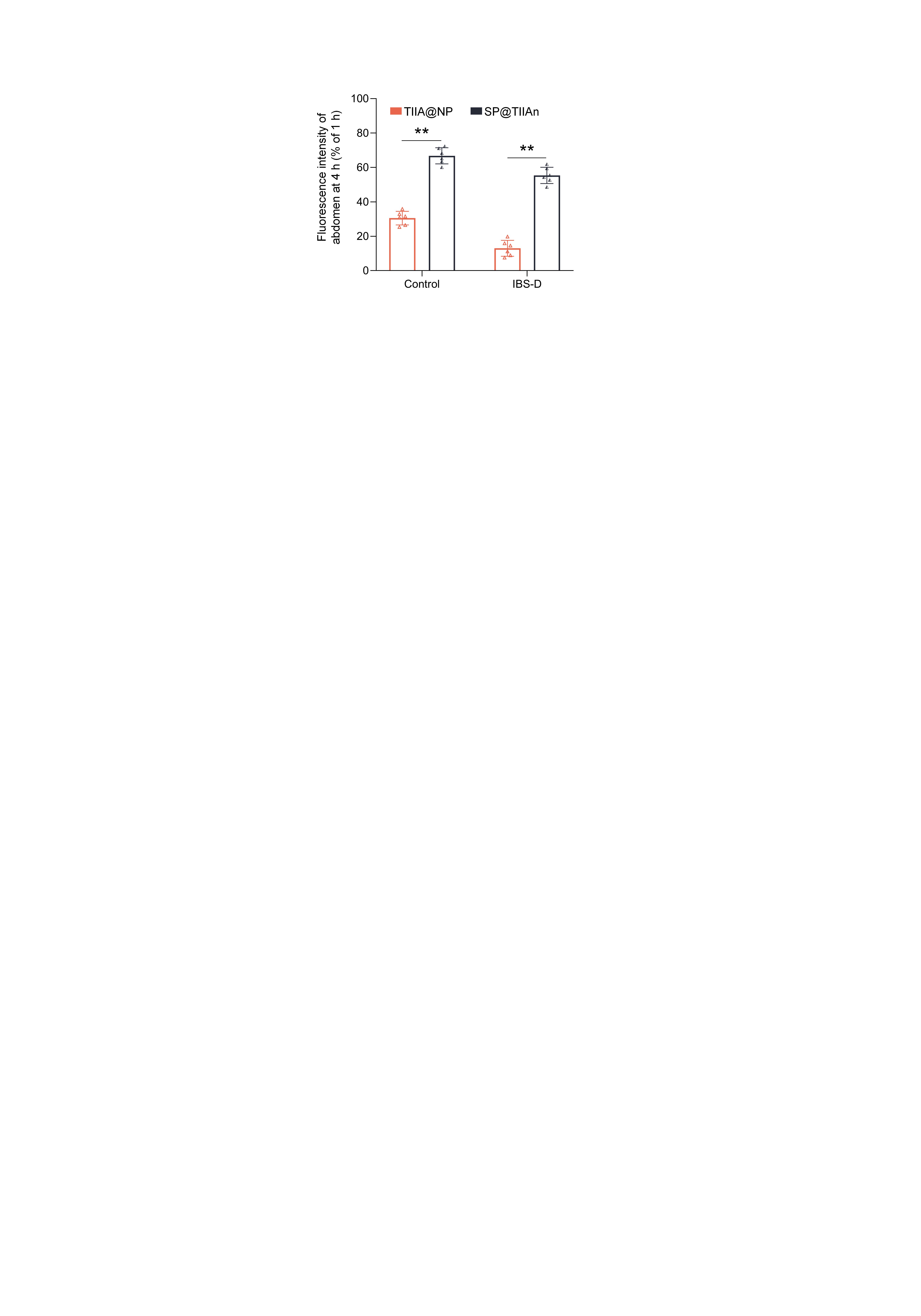


**Figure S10.** The abdominal fluorescence intensity of each group 4 hours after intragastric administration. Data are represented as the mean ± SD (n = 6). ** represents *P* < 0.01, n.s. represents no significance.


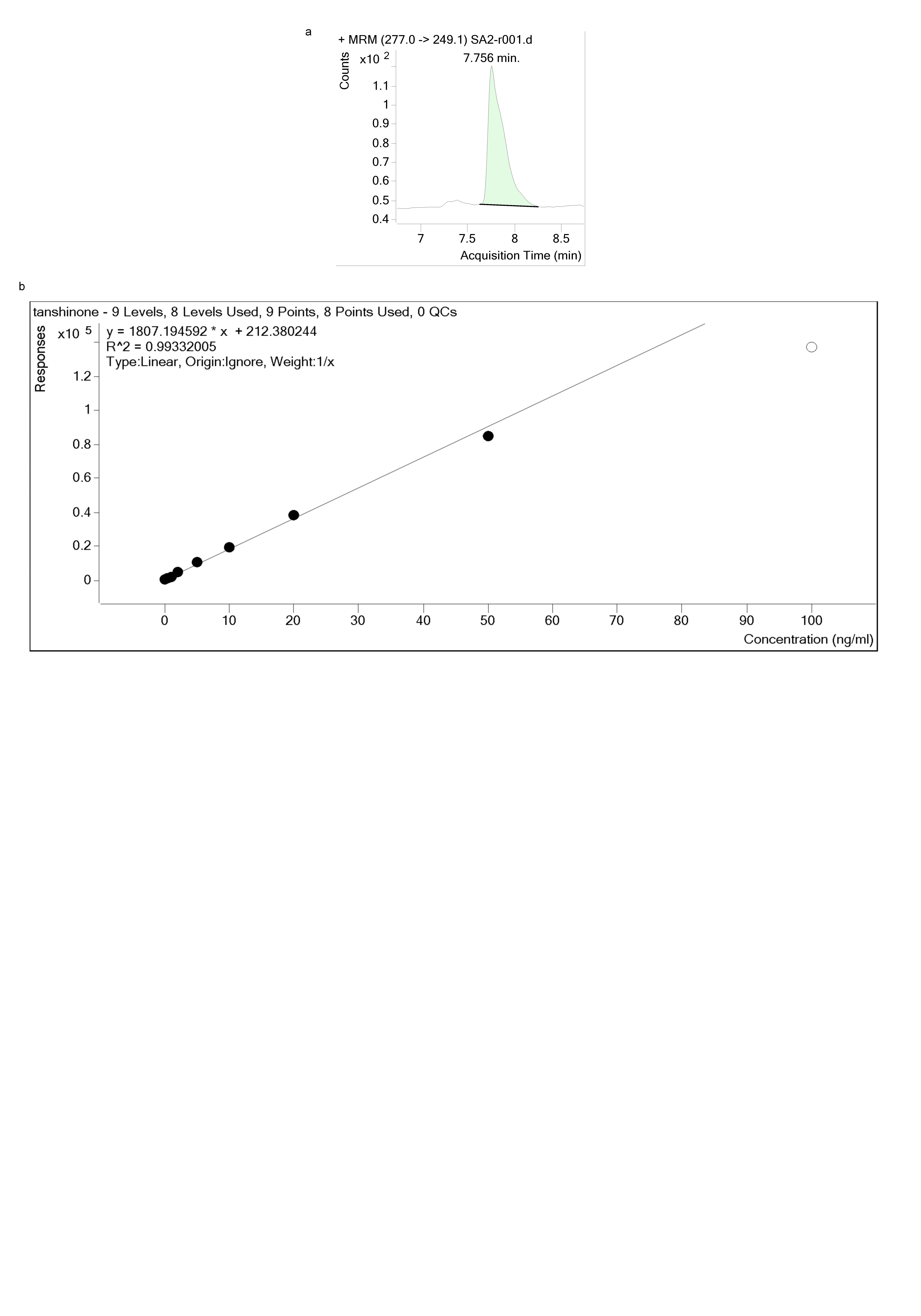


**Figure S11.** a) Representative MRM mass spectrometry chromatogram and b) standard curve of TIIA.


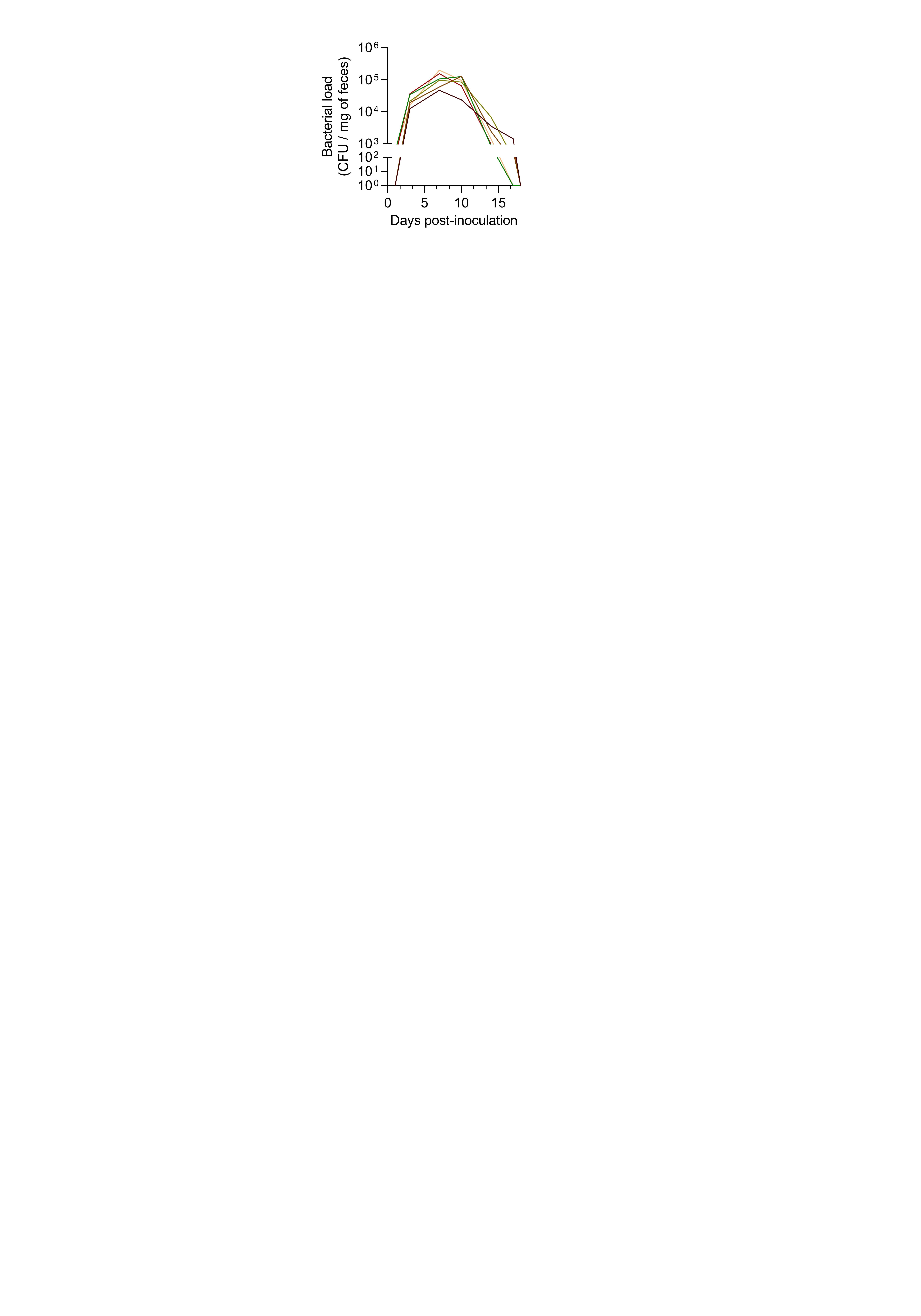


**Figure S12.** Fecal CR count after infection (n=6).


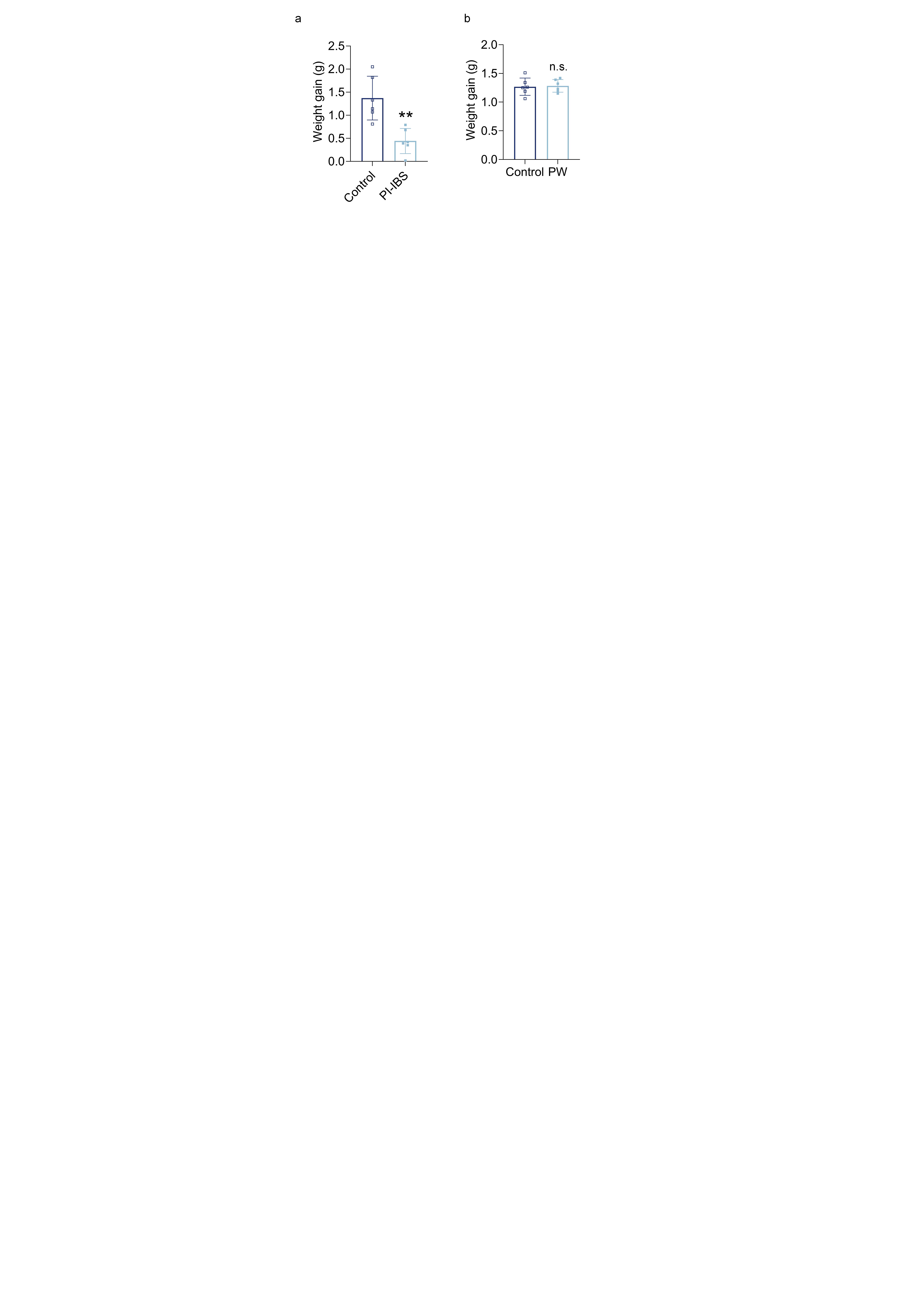


**Figure S13.** Weight gain during a) the CR infection stage and b) the WAS stage. Data are represented as the mean ± SD (n = 6). ** represents *P* < 0.01 vs. the Control group, n.s. represents no significance vs. the Control group.

**
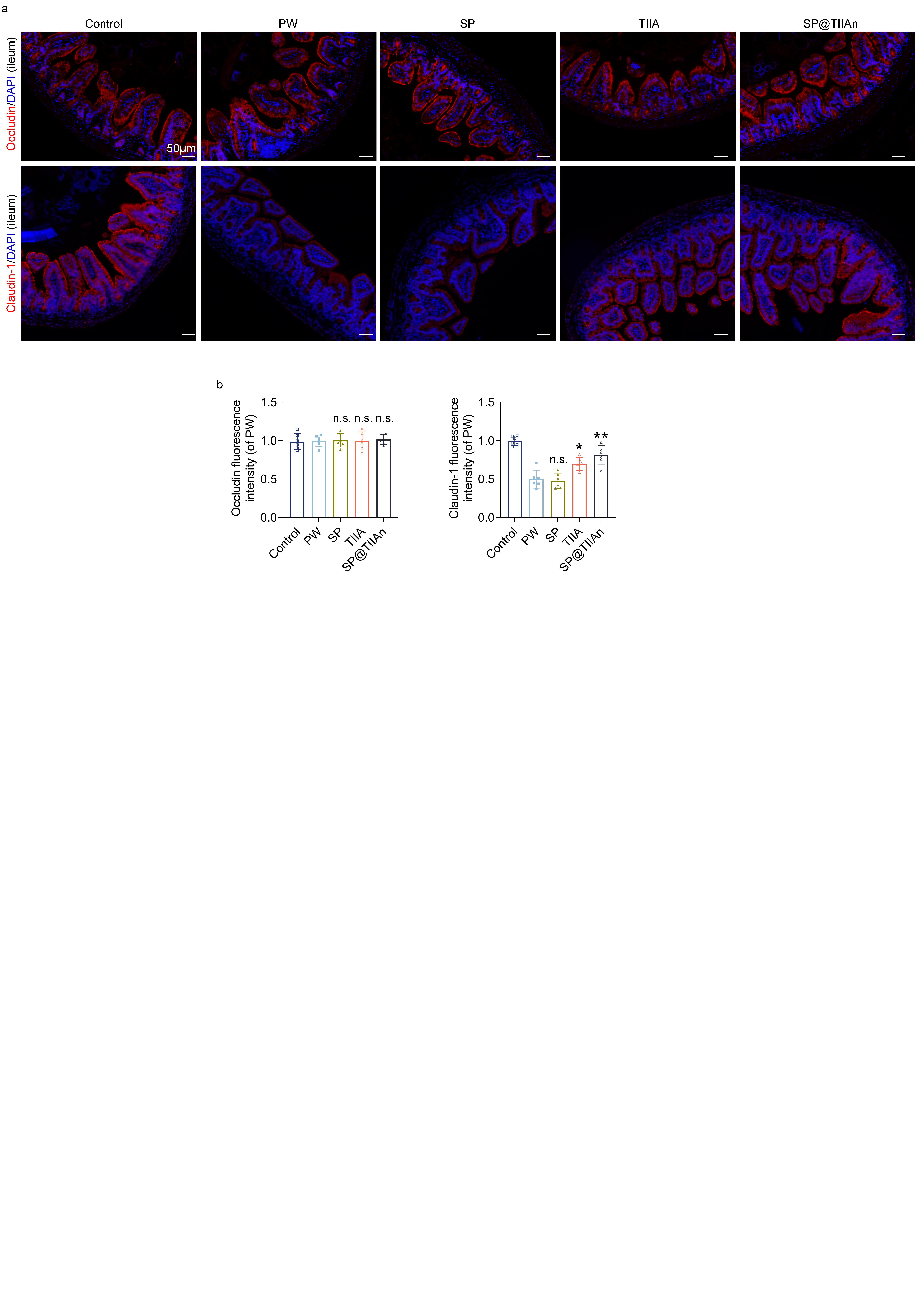
**

**Figure S14.** a) Immunofluorescence staining of ileum occludin, Claudin-1 and b) quantification. Data are represented as the mean ± SD (n = 6). * represents *P* < 0.05, ** represents *P* < 0.01 vs. the PW group, n.s. represents no significance vs. the PW group.


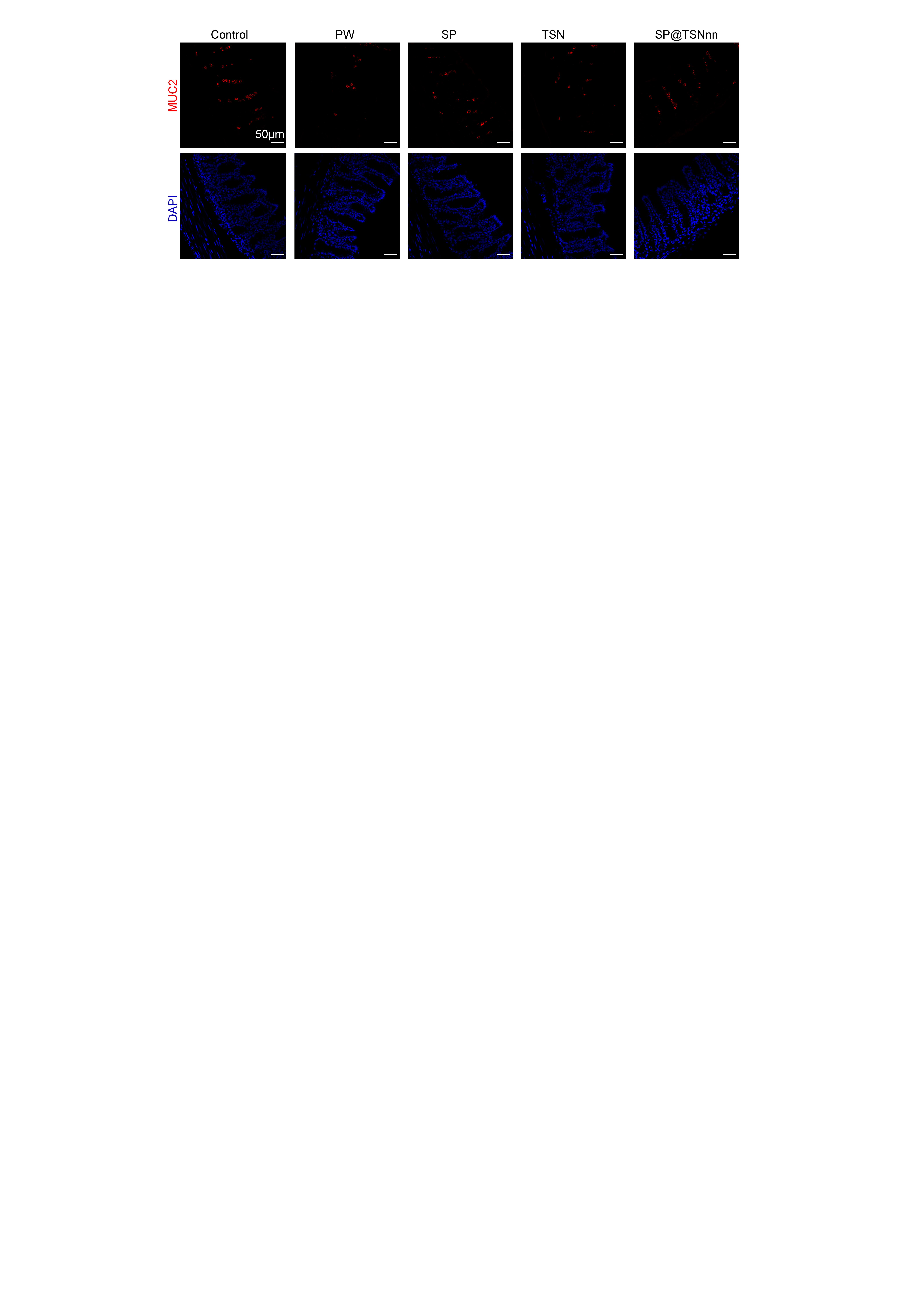


**Figure S15.** Single-channel images of Figure 4d.


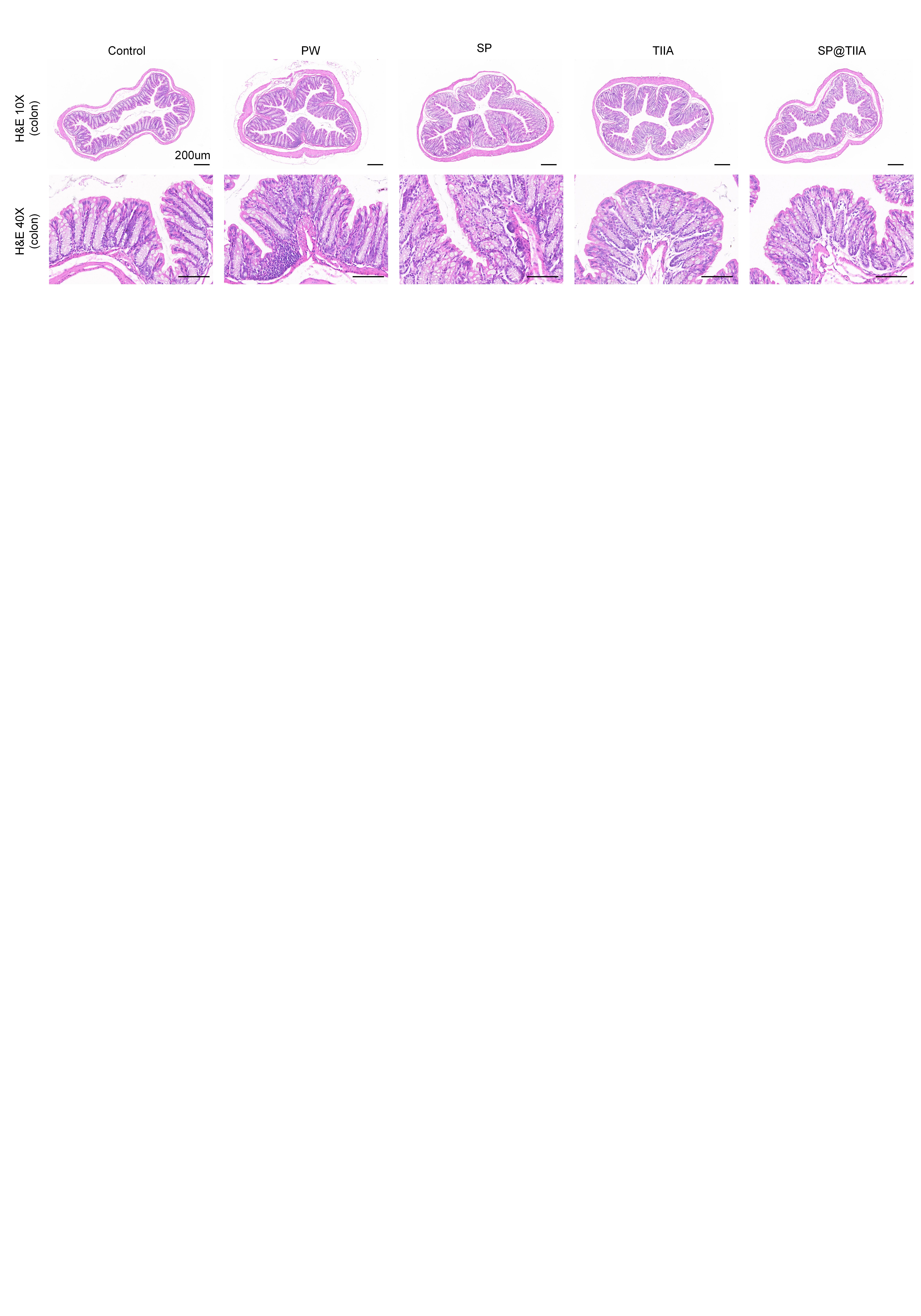


**Figure S16.** H&E staining staining of colon.


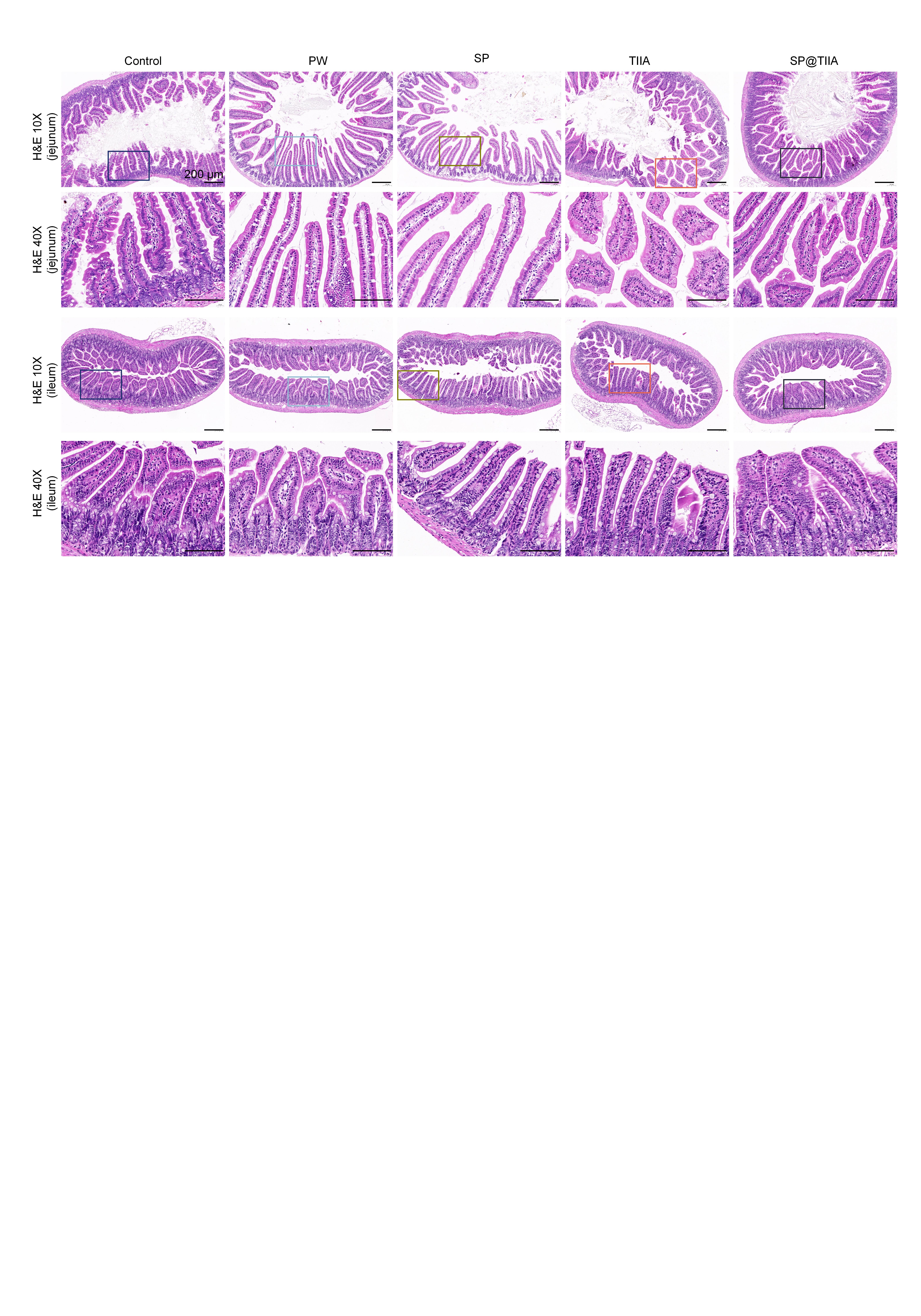


**Figure S17.** H&E staining staining of small intestine.

**
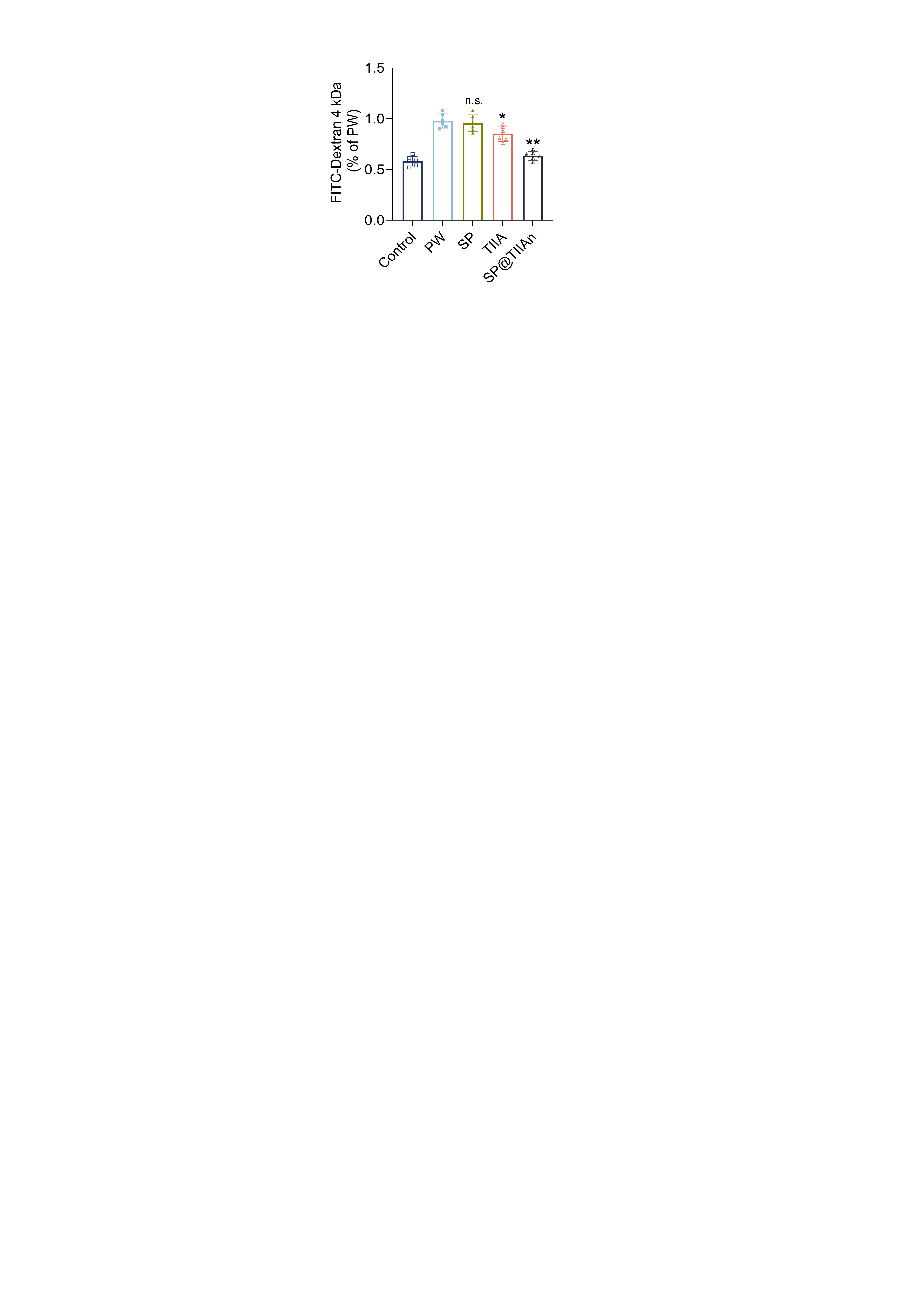
**

**Figure S18.** Intestinal permeability evaluated by serum concentration of 4 kDa FITC-Dextran. Data are represented as the mean ± SD (n = 6). * represents *P* < 0.05, ** represents *P* < 0.01 vs. the PW group, n.s. represents no significance vs. the PW group.

**
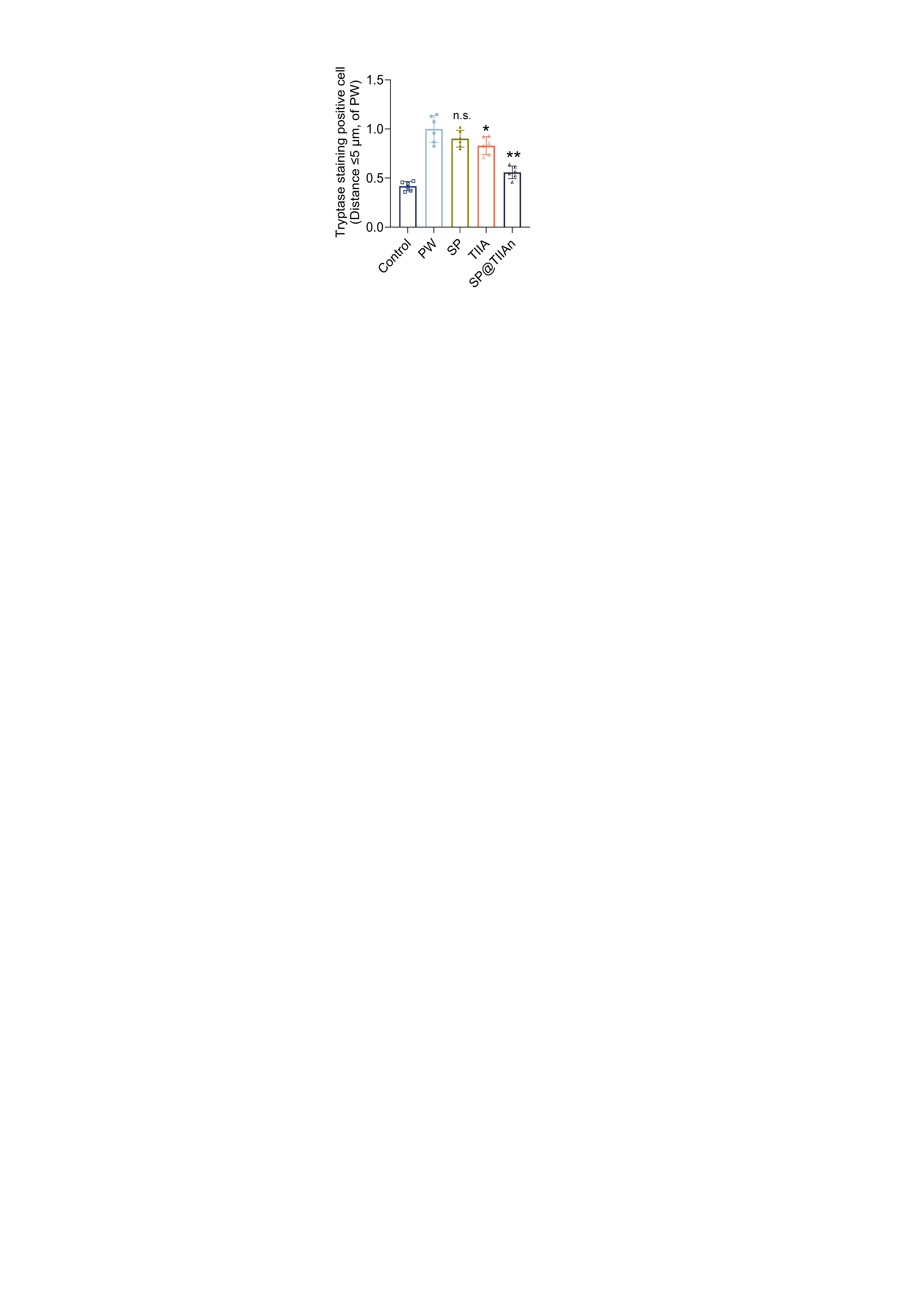
**

**Figure S19.** Tryptase-positive cells less than 5 μm from nerve fibers. Data are represented as the mean ± SD (n = 6). * represents *P* < 0.05, ** represents *P* < 0.01 vs. the PW group, n.s. represents no significance vs. the PW group.

**
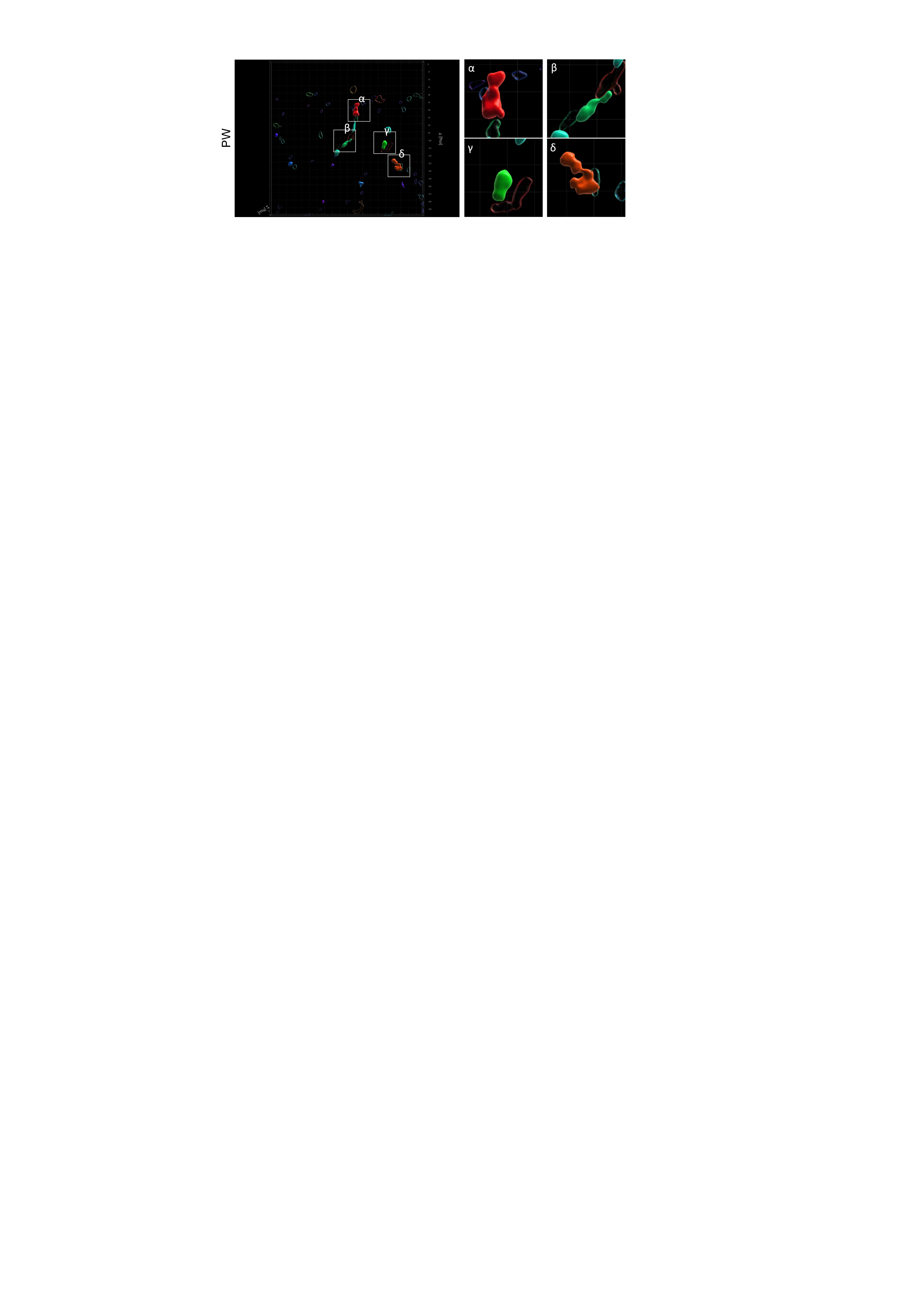
**

**Figure S20.** 3D reconstruction of mast cells and nerve fibers in PW group (Solid: mast cells, transparent: nerve fibers).

**
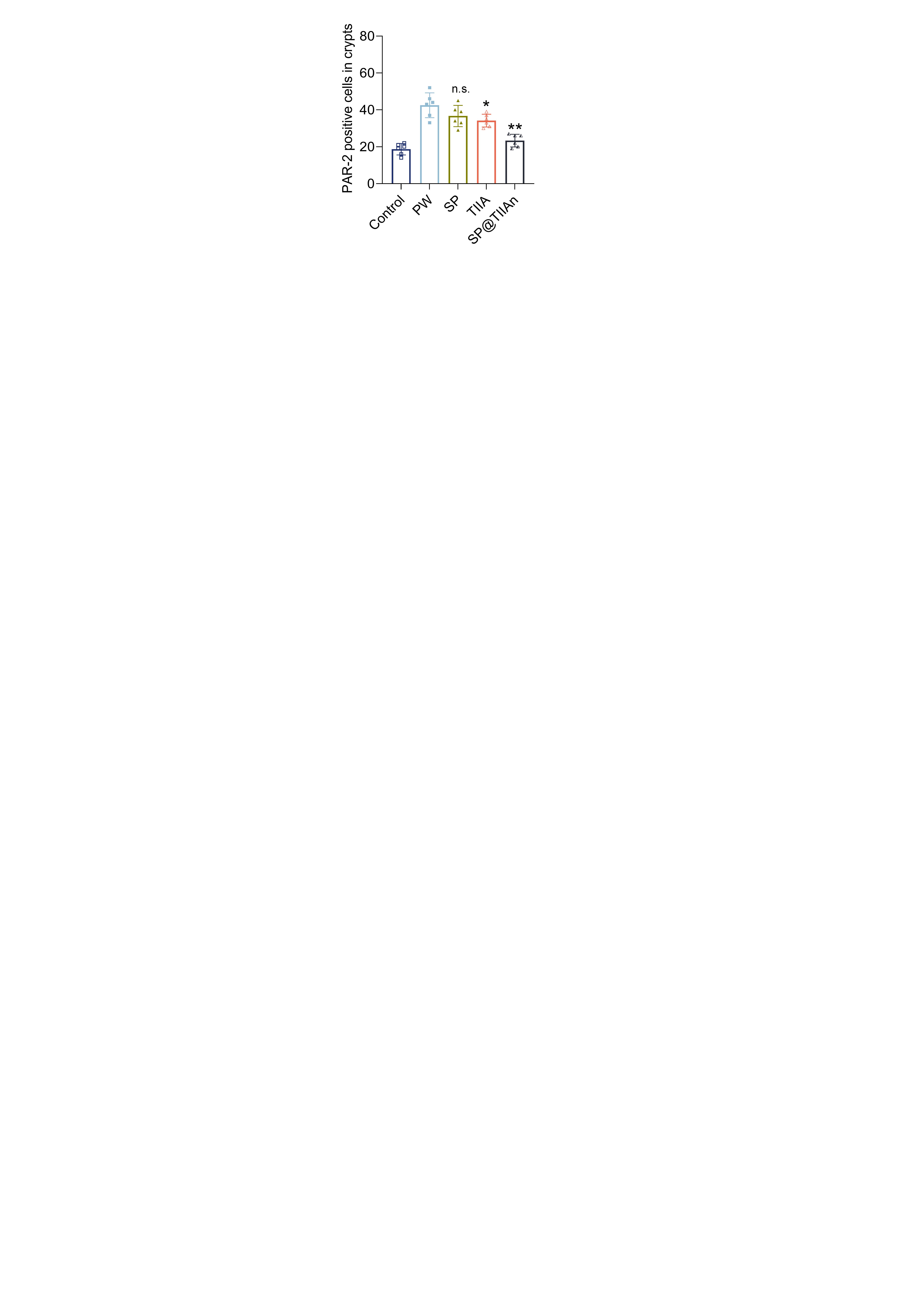
**

**Figure S21.** Number of PAR-2-positive cells. Data are represented as the mean ± SD (n = 6). * represents *P* < 0.05, ** represents *P* < 0.01 vs. the PW group, n.s. represents no significance vs. the PW group.


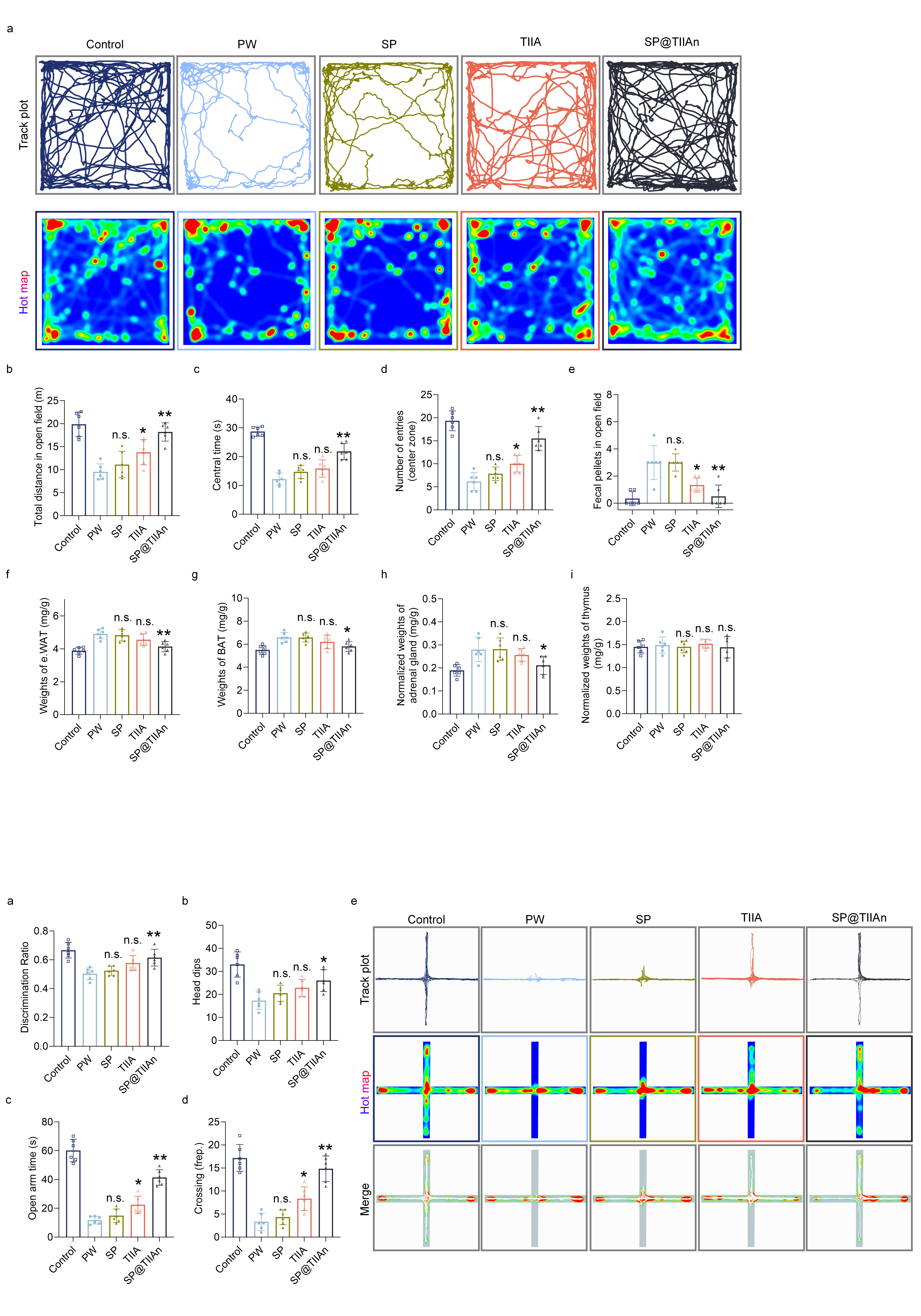


**Figure S22.** a) Discrimination ratio of NOR. b) Number of times the mice explored the hole in the hole-board experiment. c) Time spent in open arm time and d) crossing frequency and e) movement tracking images and heatmaps of mice in the EPM. Data are represented as the mean ± SD (n = 6). * represents P < 0.05, ** represents P < 0.01 vs. the PW group, n.s. represents no significance vs. the PW group.


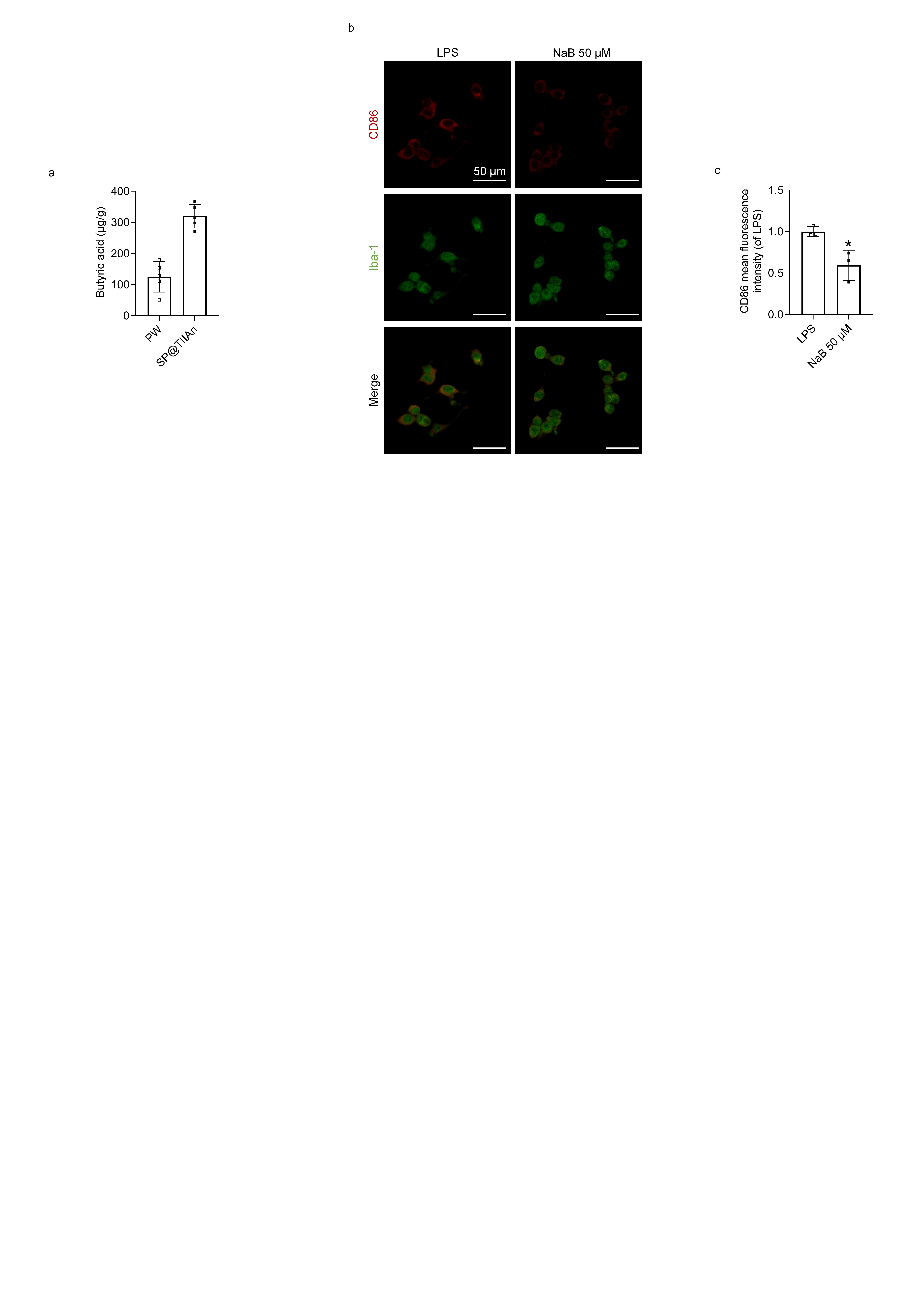


**Figure S23.** a) Butyric acid content in different groups of feces (n = 5). b-c) CD86 immunofluorescence and mean fluorescence Intensity of CD86. Data are represented as the mean ± SD (n = 3). * represents *P* < 0.05 vs. the LPS group.


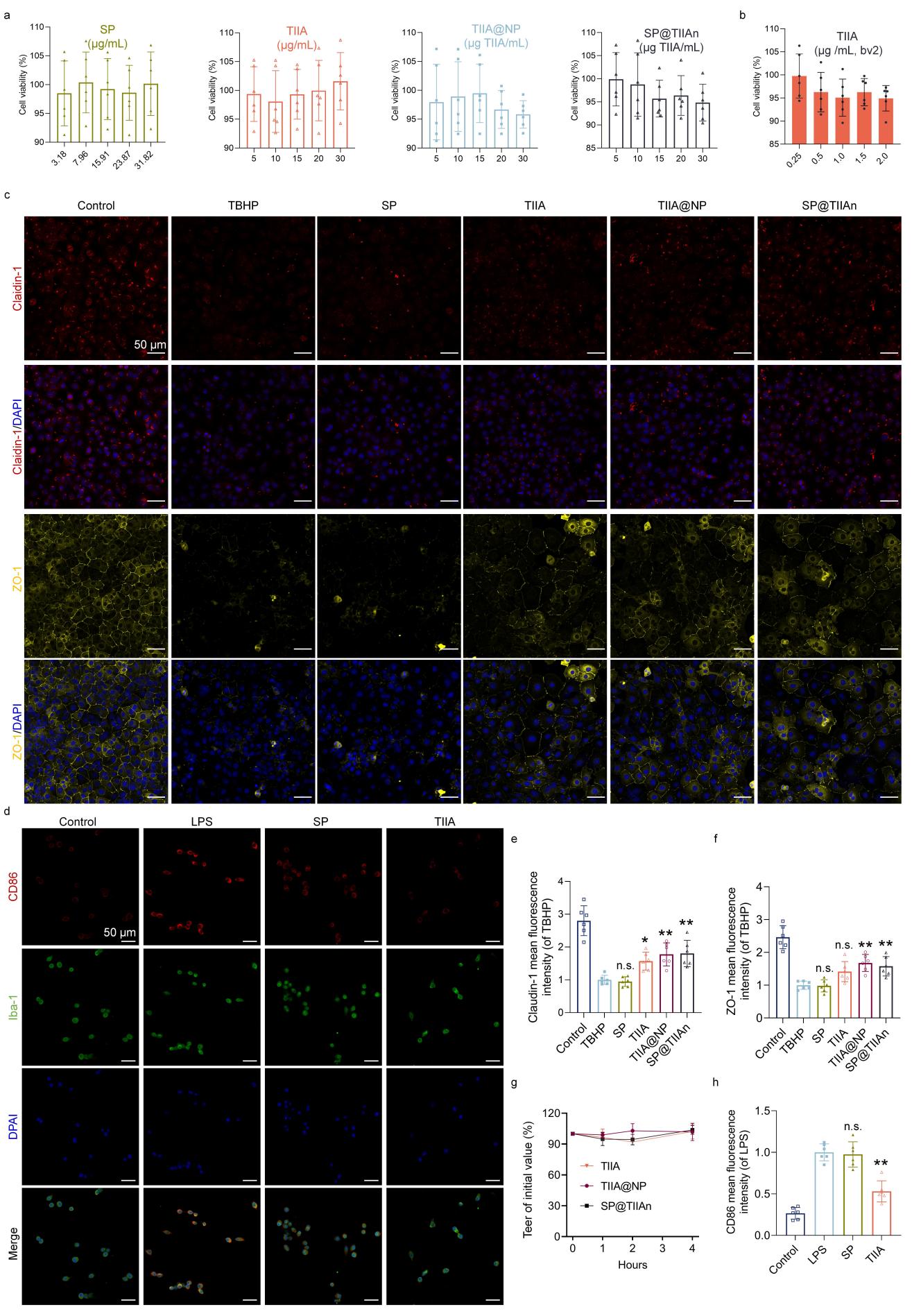


**Figure S24.** a) Cell ciability of IEC-6 cells after 24 h incubation with different concentrations of SP, TIIA, TlIA@NP, SP@TIIAn (n=6). b) Cell ciability of bv2 cells after 24 h incubation with different concentrations of TIIA (n=6). c) ZO-1 and Claudin-1 immunofluorescence staining of IEC-6 cells. d) Immunofluorescence staining for CD86 (red) and Iba-1 (green) for each group. e-f) Quantification of fluorescence intensity of Claudin-1(e) and ZO-1 (f). g) Teer values after different treatments (n=3). h) Fluorescence intensity of CD86 (n=6). Data are represented as the mean ± SD. * represents P < 0.05, ** represents P < 0.01 vs. the TBHP or LPS group, n.s. represents no significance vs. the TBHP or LPS group.
